# A single-cell polony method reveals low levels of infected *Prochlorococcus* in oligotrophic waters despite high cyanophage abundances

**DOI:** 10.1038/s41396-020-00752-6

**Published:** 2020-09-11

**Authors:** Noor Mruwat, Michael C. G. Carlson, Svetlana Goldin, François Ribalet, Shay Kirzner, Yotam Hulata, Stephen J. Beckett, Dror Shitrit, Joshua S. Weitz, E. Virginia Armbrust, Debbie Lindell

**Affiliations:** 1 Faculty of Biology, Technion – Israel Institute of Technology, Haifa, 3200003 Israel; 2 School of Oceanography, University of Washington, Seattle, WA 98195 USA; 3 School of Biological Sciences, Georgia Institute of Technology, Atlanta, GA 30332 USA; 4 School of Physics, Georgia Institute of Technology, Atlanta, GA 30332 USA

**Keywords:** Microbial ecology, Microbial biooceanography, Molecular ecology, Microbial ecology, Microbial biooceanography

## Abstract

Long-term stability of picocyanobacteria in the open oceans is maintained by a balance between synchronous division and death on daily timescales. Viruses are considered a major source of microbial mortality, however, current methods to measure infection have significant methodological limitations. Here we describe a method that pairs flow-cytometric sorting with a PCR-based polony technique to simultaneously screen thousands of taxonomically resolved individual cells for intracellular virus DNA, enabling sensitive, high-throughput, and direct quantification of infection by different virus lineages. Under controlled conditions with picocyanobacteria-cyanophage models, the method detected infection throughout the lytic cycle and discriminated between varying infection levels. In North Pacific subtropical surface waters, the method revealed that only a small percentage of *Prochlorococcus* (0.35–1.6%) were infected, predominantly by T4-like cyanophages, and that infection oscillated 2-fold in phase with the diel cycle. This corresponds to 0.35–4.8% of *Prochlorococcus* mortality daily. Cyanophages were 2–4-fold more abundant than *Prochlorococcus*, indicating that most encounters did not result in infection and suggesting infection is mitigated via host resistance, reduced phage infectivity and inefficient adsorption. This method will enable quantification of infection for key microbial taxa across oceanic regimes and will help determine the extent that viruses shape microbial communities and ecosystem level processes.

## Introduction

The marine unicellular picocyanobacteria, *Prochlorococcus* and *Synechococcus*, are the numerically dominant autotrophs in vast regions of the oceans [1, 2] and account for a major portion of primary production in oligotrophic waters [3–6]. In subtropical gyres, *Prochlorococcus* dominates the autotrophic community, and both *Prochlorococcus* and *Synechococcus* abundances are stable over long timescales [3] despite daily synchronous cell division [5, 7, 8]. Thus, picocyanobacteria mortality is likely similar in magnitude to growth and closely coupled to diel rhythms in cell division [7]. However, the extent to which different mortality agents kill marine *Prochlorococcus* and *Synechococcus* is unclear yet is essential to understanding the movement of organic matter through open ocean ecosystems.

In the surface ocean, viruses typically outnumber their microbial hosts by 1- to more than 100-fold [9] and modulate microbial abundance and diversity, evolution, and the fate of microbial organic matter [10–12]. Viral infection is assumed to be a significant source of mortality, estimated to kill between 10 and 40% of marine microbes daily [10, 11, 13–15], although reported estimates range from 0 to over 200% [16–25]. In particular, estimates of infection for marine picocyanobacteria have been reported to be highly variable, ranging from below 0.005% [18] to 46% [24]. It is unclear whether this range of estimates reflects true biological variability or is the result of methodological imprecision. The difficulty of constraining estimates of viral infection in the environment hinders a quantitative understanding of the extent to which viruses influence microbial communities and ecosystem level processes.

Current methods assess virus-mediated mortality with either direct or indirect approaches (Supplementary Table 1). Indirect approaches rely on measurements that are proxies for infection such as the production of free viruses [20] or the disappearance of microbial cells [26, 27]. A suite of direct approaches measures virus nucleic acids or capsids inside cells with either molecular or microscopic tools (Supplementary Table 1). All methods that quantify virus-mediated mortality rely on assumptions to convert measurements to mortality estimates. These assumptions are often unconstrained for natural populations, are highly debated [16, 18], and can greatly affect final mortality estimates [18]. Furthermore, most methods have limited taxonomic resolution in identifying the types of viruses responsible for infection, such that the understanding of the functional impact of viral lineages lags significantly behind the rapid rate of discovery of new lineages enabled, in large part, by metagenomics [28, 29]. For example, *Synechococcus* and *Prochlorococcus* are infected by several different lineages of viruses [18, 30–32], but the relative contribution of these different types to picocyanobacteria mortality is largely unknown. Thus, despite the numerous methods developed over the years, no direct method exists that is taxonomically resolved, sensitive and accurate, and allows for high-throughput quantification of viral infection of environmental microbes.

Here we describe the adaption of the solid-phase polony PCR method [33] for the direct quantification of viral infection. This method enables the detection of virus DNA inside cells and can be adapted to target viruses at different taxonomic levels, from single virus genotypes to genetically diverse virus families. We developed the method under controlled laboratory conditions for marine picocyanobacteria infected by T4-like and T7-like cyanophages, two of the most abundant cyanophage lineages in the ocean [33, 34]. The method was then used to quantify *Prochlorococcus* infection at high temporal resolution over seven diel cycles in the upper mixed layer of the North Pacific Subtropical Gyre (NPSG) to understand the contribution of viruses to the daily mortality of *Prochlorococcus*. We found that viruses infected only a small fraction of the *Prochlorococcus* population (averaging 0.79%) despite high abundances of cyanophages and that infection incidence oscillated over the diel cycle. Widespread use of this method will forge quantitative links between viral infection and its impact on microbial communities and the cycling of organic matter through the microbial loop.

## Materials and methods

We developed, validated and implemented the iPolony method for determining percent infection by cyanophages. The iPolony method is based on the detection of viral DNA inside cyanobacteria. Infection experiments were performed using model cyanophage-picocyanobacteria systems under controlled conditions, which tested the ability of the iPolony method to detect infection throughout the infection cycle and to distinguish different levels of infection. We employed the method to measure infection of *Prochlorococcus* over 7 diel periods in the surface waters of the North Pacific Subtropical Gyre. These data were then used to inform ecological models to estimate the contribution of viruses to *Prochlorococcus* mortality during this sampling period (Supplementary methods).

### Infection experiments

Phage growth curves were performed for the T7-like podovirus, Syn5, on *Synechococcus* sp. strain WH8109 and for the T4-like myovirus, S-TIM4, on *Prochlorococcus* sp. strain MIT9515. For growth conditions and lysate preparation, see Supplementary text. Cultures were infected during the mid-to-late log phase of cyanobacterial growth at cell concentrations of 4.6–5.7 × 10^7^ cells ml^−1^ for *Synechococcus* and 2–2.3 × 10^8^ cells ml^−1^ for *Prochlorococcus* and at a multiplicity of infection (MOI) of three infective viruses per host unless otherwise stated. Samples were collected periodically throughout the infection cycle for quantification of viruses in the extracellular medium, viral DNA replication, host DNA degradation, and quantification of percent infection by the iPolony method. Uninfected control cultures were supplemented with the same volume of growth medium rather than virus lysate. Infected and uninfected cultures were then incubated under cyanobacterial growth conditions. The Syn5 infections were synchronized by 10,000-fold dilution of the infection mix with culture medium at 5 min post infection for percent infection measurements by iPolony, but not for the other measurements. S-TIM4 infections were synchronized at 1 h post infection by pelleting cells, removing the phage-containing supernatant, washing the pellet with sterile seawater, and resuspending in fresh medium. For quantification of viruses in the extracellular medium, the concentration of infective viruses in the lysate was determined for Syn5 using the plaque assay by plating serial dilutions of the <0.2 µm fraction of culture on lawns of host cells. The lawns were incubated under standard growth conditions and plaques were enumerated from 1 day to 2 weeks after plating. For S-TIM4, qPCR was used to determine the timing of release of viruses into the extracellular medium because plaque formation on lawns of MIT9515 is not robust. Synchronization by pelleting was used for S-TIM4 to reduce the high background of non-infective viruses in the lysate to enable accurate detection of the timing of newly released viruses by qPCR. For intracellular virus and host genomic DNA quantification, cultures were filtered onto a 0.2 μm polycarbonate filter, washed with sterile seawater and preservation solution (10 mM Tris, 100 mM EDTA, 0.5 M NaCl; pH 8) to remove extracellular viruses, and flash frozen in liquid nitrogen. The amount of intracellular host and viral gDNA was analyzed by qPCR as outlined in Lindell et al. [35]. The extent of viral infection was quantified using the iPolony method (described below). For the latter, infection was stopped by fixation with 0.1% glutaraldehyde at room temperature in the dark for 15 min. Fixed samples were flash frozen in liquid nitrogen and stored at −80 °C.

To test the ability of the polony method to detect differences in the extent of infection, cultures of *Synechococcus* sp. strain WH8109 were infected with the Syn5 T7-like podovirus at different MOIs. Infection experiments were performed in 1 ml volumes and samples were collected after maximal adsorption which was empirically determined to be 30 min after infection for Syn5 [36]. These samples were diluted 50-fold with medium, fixed, and frozen prior to analysis. The percent infection from the experiments conducted at different MOIs was also assessed by a lysis-based flow-cytometric sorting assay [37]. Briefly, infected samples were diluted 1000-fold in growth medium, and single cells were sorted by flow cytometry into individual wells of 96-well plates that contained exponentially growing host cell culture. Cells from uninfected cultures were sorted into plates as a control. The plates were incubated under cyanobacterial growth conditions. If the deposited cell is infected and releases viruses, the culture in the well undergoes lysis. After culture lysis, the percent infection was determined from the proportion of cleared wells.

### Flow-cytometric cell sorting and analysis

Flow cytometric sorting and analysis was used to purify, concentrate, and quantify cells in laboratory infection experiments and from environmental samples. Samples were amended with 1 µm yellow-green beads as an internal reference. Sorting was performed on a Becton Dickinson (BD) Influx cytometer, except for experiments involving cultured *Synechococcus* which were sorted on a BD FACSAria-IIIu cell sorter. Phosphate buffered saline (137 mM NaCl, 2.7 mM KCl, 10 mM Na_2_HPO_4_, 1.8 mM KH_2_PO_4_, pH 7.4) was used as the sheath fluid. The FACSAria was equipped with a 488 nm laser and an 85 µm nozzle tip. The Influx was equipped with a small particle detector (a high numerical aperture microscope lens, focusing pinhole, and photomultiplier tube) and either a 488 nm laser or both a 488-nm and a 457-nm laser, and a 70-µm nozzle tip. With this configuration, the Influx cytometer enabled detection of the small and dimly fluorescent surface populations of *Prochlorococcus* and yielded high concentrations of sorted cells (700–1000 cells μl^−1^) necessary for downstream analyses [38]. *Synechococcus* was gated and sorted based on orange fluorescence (phycoerythrin containing cells) and size based on forward scatter. *Prochlorococcus* was gated and sorted based on red fluorescence (chlorophyll containing cells) and size based on forward scatter. Hierarchical gating based on orange then red fluorescence was employed to discriminate between *Prochlorococcus* and *Synechococcus* from mixed communities. Sorting was performed in the 1.0 drop purity mode. After sorting, sorted cell concentrations were quantified on either the Influx cytometer or on a BD LSR-II Analyzer (the latter was used only for cultured *Synechococcus*) and checked for purity of *Prochlorococcus* or *Synechococcus* based on size and fluorescence. Cell populations were analyzed using the FCS Express 6 or FACSDiva v8 software.

### Polony procedure for infected cells

Polony gels containing sorted cells were cast into a sterile 40-μm deep well of Bind-Silane pretreated microscope slides (Thermo Fisher Scientific). All samples described in this study used degenerate primers except for experiments of cell lysis conditions which used specific primers (see Supplementary text for reaction conditions). The gel mix contained 10% acrylamide and 10 or 20 μM 5′-acrydite-modified primer targeting T4-like [34] and T7-like cyanophages [33], respectively, and up to 8 µl sorted cells, which ranged between ~200–10,000 and 8000–10,000 cells per gel for lab and field samples, respectively. The number of cells input was empirically determined to be within the bounds of 5–2000 polonies per slide based on the lower limit of accurate detection and the upper limit where polonies are not overlapping. Thus, the number of cells per gel should be optimized depending on infection levels and has a lower limit of detection of 0.05% when 10,000 cells are interrogated per slide. Note that for the T4-like infection assay, cells are not treated with heat and EDTA as in the polony method for free T4-like cyanophage [34] as non-encapsidated virus DNA is present inside cells throughout the infection cycle [39]. Gels were polymerized for 30 min under argon gas. Next, gels were washed twice with sterile MilliQ water and once with 0.025% Tween-20 to remove residual acrylamide and air-dried. Diffuse-in mix (20 µl) containing the other PCR reagents were applied to the gel immediately after drying. The mix contained 1x *Taq* buffer, 0.25 mM deoxyribonucleotide triphosphate (dNTP) mix, 10 µM non-modified primer, and 0.67 U/µl Jumpstart *Taq* polymerase (Sigma-Aldrich). For a list of primers and probes used in this study, refer to Supplementary Table 2 and Supplementary text. All pre-PCR processes described above were performed in a sterile environment.

In cyanophage growth experiments and select environmental samples (Supplementary text), sorted cells were filtered through a 0.2 μm syringe filter (Acrodisc HT Tuffryn Membrane, Pall Corp.), and the filtrate was run under the same conditions as sorted cells to assess the abundance of co-sorted free viruses (Supplementary text).

Prior to thermal cycling, gels were heated to 94 °C for 15 min to lyse cells, followed by incubation at 25 °C for 15 min to allow the entry of PCR reagents and enzymes into lysed cells. Thermal cycling consisted of 50 cycles of denaturation at 94 °C for 45 s, annealing at 50 °C for 45 s for T7-like cyanophages [33] or a stepped 0.3 °C gradient from 35  to 50 °C for T4-like cyanophages [34] and elongation at 72 °C for 2 min. The procedure ended with a 6 min elongation step at 72 °C.

After thermal cycling, PCR amplicons in the gels were denatured in a solution of 70% formamide, 150 mM NaCl, and 15 mM trisodium citrate for 30 min at 70 °C and then washed once with MilliQ water and twice with buffer E (10 mM Tris pH 7.5, 50 mM KCl, 2 mM EDTA pH 8, 0.01% Triton X-100). Hybridization mix (900 mM NaCl, 60 mM NaH_2_PO_4_, 6 mM EDTA, 0.01% Triton X-100) (130 µl) containing Cy5 and/or Cy3-modified probes (Supplementary Table 2) at concentrations of 0.6 or 1.2 μM for Cy5 and Cy3 probes, respectively, was applied to the gel. The gel was heated to 94 °C for 6 min and probes were annealed at 42 °C for 30 min for degenerate probes and at 55 °C for 15–20 min for specific probes. The probes are internal to the PCR amplicon to ensure detection of specific amplicons. Hybridized gels were washed with buffer E three times for 30 min and once overnight at room temperature to remove excess probe.

Gel scanning was performed with a GenePix 4000B or 4000A microarray scanner (Axon Instruments) using GenePix Pro software with the 635 nm laser used for Cy5 detection and the 550 nm laser for Cy3 detection. Polonies were counted manually using ImageJ software. All samples were analyzed in duplicate, at a minimum.

Percent infection was determined by normalizing the number of polonies identified in a gel to the number of input cells. Two corrections were then applied as necessary. First, the number of polonies that resulted from co-sorted viruses in the <0.2 µm filtrate was subtracted from the total number of polonies that contained cells and co-sorted viruses when measured empirically. For environmental samples in which co-sorted viruses were not analyzed, if T7-like cyanophage and T4-like cyanophage concentrations in the water column exceeded the co-sorting threshold of 5 × 10^5^ and 3 × 10^5^ ml^−1^, respectively, percent infection values were multiplied by 0.918 or 0.805, respectively, to correct for co-sorted viruses (see Supplementary material). Second, corrections were made to account for differing detection efficiencies throughout the latent period, which is the length of time in the infection cycle prior to new phage progeny release and cell death. Three bins were used to represent detection efficiencies of 25, 55, and 86%, based on empirical findings at different stages of the infection cycle corresponding to the periods prior to, during, and after virus genome replication and were weighted for their relative duration during the latent period (see Supplementary methods). Bootstrapping with resampling (10,000 iterations) was employed to adjust iPolony measurements for the uncertainties in the stage of infection in natural communities. To determine the bounds of infection that were used to calculate the maximum differences in infection if all infections were perfectly synchronized, polony values were divided by 25% for an upper bound representative of all infections being prior to genome replication and 86% for a lower bound representative of all infection being after genome replication.

Samples were able to be flash frozen and stored for at least 1-year post collection without any effect on infected cell abundances (Supplementary Fig. 1). Once thawed, however, samples must be processed immediately as storing sorted cells longer than 24 h at 4 or −80 °C resulted in loss of infected cells (Supplementary Fig. 2). For further information on the storage and stability of samples over time, refer to the Supplementary text.

### Diel sample collection and analysis

Samples were collected in the North Pacific Subtropical Gyre following a Lagrangian water mass near Station ALOHA aboard the *RV Kilo Moana* from July 26 to August 3, 2015. Details about the cruise track design and execution can be found in Wilson et al. [40]. Samples were collected for viral abundance and infection every 4 h from 15 m depth in 12 L Niskin bottles attached to a conductivity-temperature-depth rosette. Water was prefiltered through a 20-μm mesh. For infected cell analyses, 40 ml samples were immediately fixed with electron microscopy grade glutaraldehyde (0.125% final concentration), incubated for 30 min in the dark at 4 °C, frozen in liquid nitrogen, and stored at −80 °C. For free virus abundances, 40 ml samples were filtered through a 0.22 µm syringe top filter (Millex-GV PDVF, Millepore). For samples used in polony analyses of free cyanophages, the filtrate was frozen without fixation at −80 °C. For samples used in virus-like particle analyses, formaldehyde (2% final concentration) was added to the filtrate prior to freezing in liquid nitrogen and storing at −80 °C.

The optical properties of cyanobacterial cells were measured continuously using SeaFlow [38]. Cell abundances and size were analyzed as in Ribalet et al. [41] with the R package *popcycle v1.1*.

Abundances of viruses and infected cells were measured after the cruise in the lab. Virus-like particles (VLPs) were enumerated using epifluoresence microscopy after staining with SYBR Green [42]. Abundances of T7-like and T4-like cyanophage in the water column were analyzed as described in Baran et al. [33] and Goldin et al. [34]. Quantification of viral infection using the iPolony method was performed 2 years after collection as described above. Co-sorted free virus abundance was tested empirically for the sample with the highest number of free cyanophages and was below the limit of accuracy. Therefore, free phage did not contribute to percent infect and no correction was needed for this dataset. For ecological models used to calculate daily infection and encounter rates, refer to Supplementary text. Statistical analysis of infection periodicity was performed using the ‘RAIN’ package v3.10 with Bioconductor in R [43]. All other statistical analyses were performed in python using the SciPy v1.4.1 library [44].

## Results and discussion

We first describe the development of the method and its validation under controlled laboratory settings for both *Synechococcus* and *Prochlorococcus* host-phage systems. Then we apply the method to quantify the contribution of T4-like and T7-like cyanophage infection to the mortality of *Prochlorococcus* over the diel cycle in the upper mixed layer of the North Pacific Subtropical Gyre during the summer of 2015.

### Development of the iPolony method for the detection of viral infection

Our goal was to develop a sensitive, high-throughput method amendable to analysis of field samples that can assess infection by virus families of interest. The polony method (named for PCR colonies that form during the reaction) is a direct PCR-based method that detects virus DNA inside infected cells. The original polony method was developed by Mitra and Church [45] and was subsequently adapted for the enumeration of free virus particles from the two major cyanophage lineages, the T4-like cyanomyoviruses [34] and the T7-like cyanopodoviruses [33]. Here we describe further development of the method to simultaneously detect viral infection in thousands of individual cells embedded in a solid-phase gel in a high-throughput manner. We term this method for the quantification of infected cells ‘iPolony’.

**Fig. 1:**
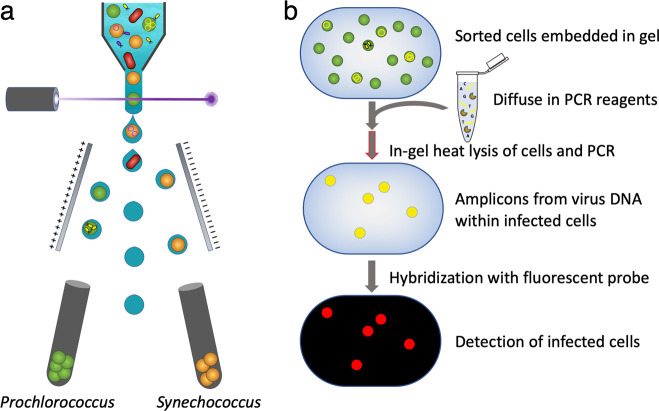
iPolony: a polony method for quantifying virally infected cyanobacteria. **a** First, *Prochlorococcus* and *Synechococcus* are sorted based on size and their autofluorescence properties using a flow cytometer from fixed samples. **b** Then, thousands of sorted cells per slide are screened for the presence of intracellular viral DNA using a solid-phase PCR polony method. Percent infection is determined based on the fraction of input cells that resulted in polonies at the end of the analysis.

The iPolony method has two steps (Fig. 1). In the first step, infection is arrested by fixation with glutaraldehyde, and target cells are isolated and concentrated (Fig. 1a). In its use here, *Prochlorococcus* and *Synechococcus* are sorted by flow cytometry based on forward angle light scattering, a proxy for cell size, and the autofluorescence of chlorophyll *a* and phycoerythrin, respectively. The concentration of cells is then quantified to calculate the number of cells analyzed in the downstream molecular analysis. In the second step, the cells are screened for the presence of intracellular virus DNA using the polony method (Fig. 1b). Polyacrylamide gels are cast with embedded sorted cells and a 5′-acrydite-modified primer to anchor the primer to the gel. PCR reagents are diffused into the gels. Degenerate PCR primers target a signature gene shared by the virus group of interest, in this case the DNA polymerase gene for the T7-like cyanopodoviruses [33] and the portal protein gene (*g20*) for the T4-like cyanomyoviruses [34]. Prior to thermal cycling, cells are permeabilized to allow amplification of virus DNA with an in-gel heat lysis step (Supplementary text, Supplementary Fig. 3). During thermal cycling, a cell that contained virus DNA results in an anchored sphere of amplification or polony. After amplification, polonies in gels are hybridized with fluorescently labeled degenerate cyanophage group-specific probes, and gels are scanned with a microarray scanner. Each individual cell is counted as infected whether there is a single or multiple virus genome copies, which enables quantification of infection in a presence–absence manner per cell. Percent infection is calculated by dividing the number of polonies by the number of cells in the gel.

### Validation of the iPolony method in controlled virus growth experiments

**Fig. 2:**
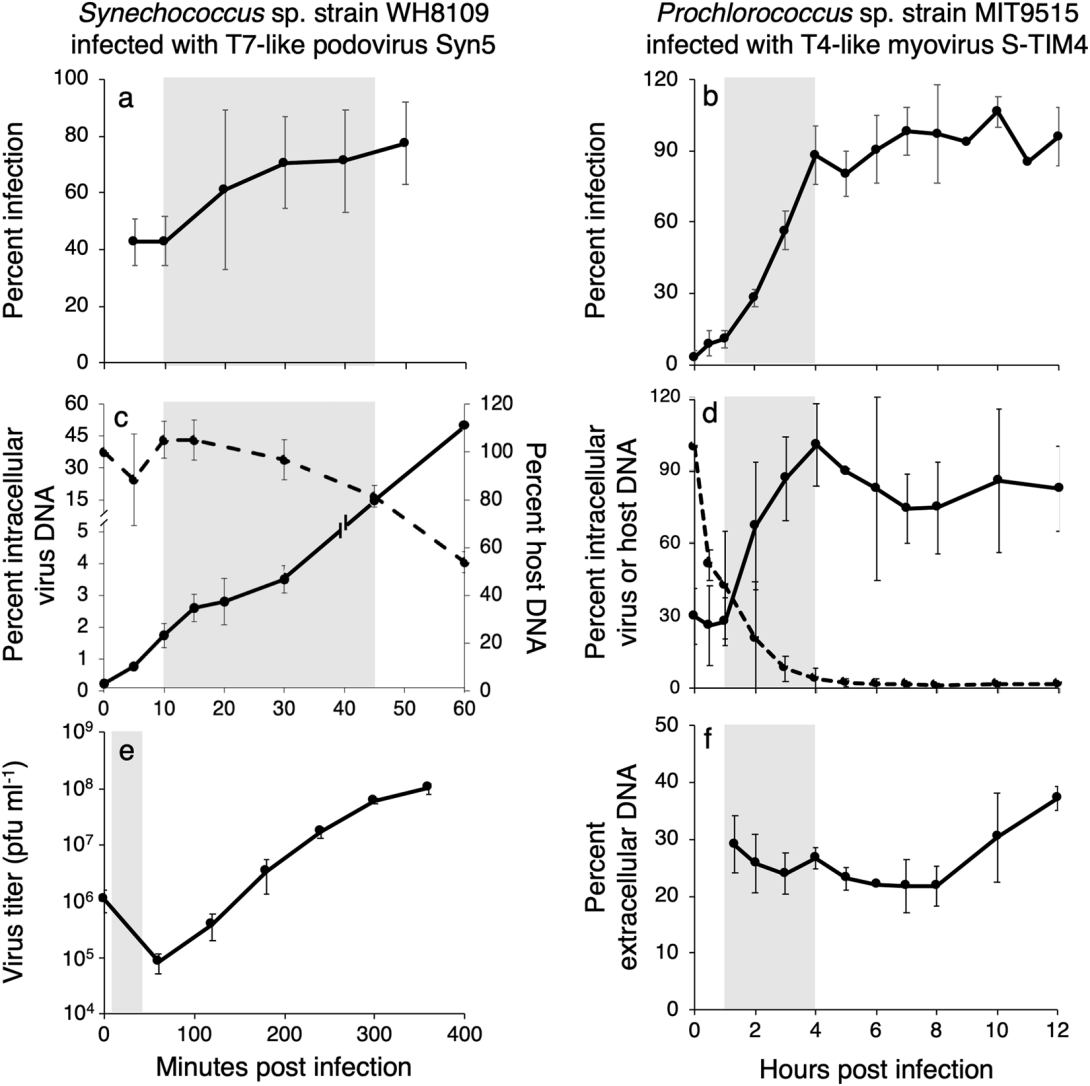
The iPolony method detects viral infection throughout the infection cycle. Cultures of *Synechococcus* sp. strain WH8109 infected by the T7-like cyanopodovirus, Syn5 (**a**, **c**, **e**) and *Prochlorococcus* sp. strain MIT9515 infected by the T4-like cyanomyovirus, S-TIM4 (**b**, **d**, **f**) at MOI = 3. **a**, **b** Percent infection was determined using the polony method over the infection cycle. **c**, **d** Virus DNA replication (solid lines) and host genomic DNA degradation (dashed lines) were assessed by qPCR in infected cultures. Host and virus DNA concentrations were normalized to initial or maximum concentrations, respectively. Shaded regions indicate the period of virus genome replication. Lysis was assessed from an increase in plaque forming units measured by the plaque assay for Syn5 (**e**) or the appearance of extracellular virus DNA measured by qPCR for S-TIM4 (**f**). Note that Syn5 infections shown in (**c**) and (**e**) were not synchronized at 5 min post infection as in (**a**) and are shown for comparison of the timing of different phases of infection. Average and standard deviation of biological triplicates are shown in all panels.

The method was first developed and tested on model lab systems under known conditions. We assessed the ability of the iPolony method to detect virus DNA inside cyanobacterial cells throughout the infection cycle. Two model systems were used: *Synechococcus* sp. strain WH8109 infected with the T7-like cyanophage, Syn5, and *Prochlorococcus* sp. strain MIT9515 infected with the T4-like cyanophage, S-TIM4. Virus DNA was detected inside cells at all stages of the infection cycle (Fig. 2). The efficiency of detection was lower prior to virus genome replication and was 43% for Syn5 and 11% for S-TIM4 (Fig. 2a–d). Detection of infected cells rose with the onset of DNA replication, reaching 86% of maximal infection levels based on host gDNA degradation, midway through genome replication for Syn5 and at the end of genome replication for S-TIM4 (Fig. 2a–d). Since more than a single phage can enter cells in these experiments, we verified that single gene copies can be detected inside cells for a single copy host gene, *rbcL*, in *Synechococcus* WH8109, as well as for *E. coli* carrying a single plasmid with a cyanophage *g20* copy (see Supplementary results and discussion). These findings indicate the method is sensitive enough to detect infection throughout the entire infection process even when a single molecule of phage DNA is present prior to genome replication and reaches maximal levels after the onset of DNA replication.

**Fig. 3:**
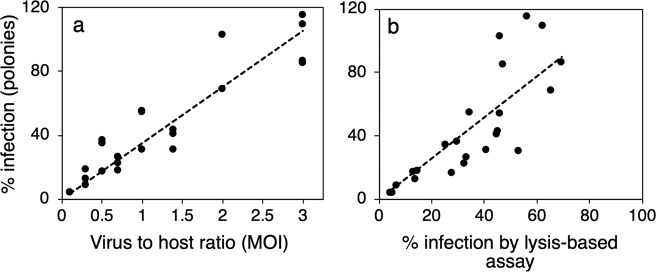
The iPolony method quantifies infection across a wide range of infection values. *Synechococcus* WH8109 was infected by the T7-like cyanophage, Syn5, at different MOIs to test the ability of the polony method to detect differences in percent infection compared to **a** the MOI in each experiment and to **b** a culture-based assay for percent infection [37]. Trend lines represent significant linear regressions between percent infection using the iPolony method and MOI values (*F* = 143.1, *R*^2^ = 0.87, *p* < 0.001) or percent infection based on the lysis-based assay (*F* = 55.60, *R*^2^ = 0.70, *p* < 0.001).

Next, we assessed whether the method could accurately detect varying levels of infection by exposing *Synechococcus* sp. strain WH8109 to different numbers of the Syn5 phage, such that the infective virus-to-host ratios (multiplicities of infection, MOIs) ranged from 0.1 to 3, spanning infection percentages that theoretically range from 10 to 95% based on encounter theory estimates (Supplementary Table 3). The percent infection determined by the iPolony method was significantly and positively correlated with the MOI (Fig. 3a) (*F* = 143.1, *R*^2^ = 0.87, *p* < 0.001) and with values obtained using a lysis-based culture-dependent assay [37] (Fig. 3b) (*F* = 55.60, *R*^2^ = 0.70, *p* < 0.001). Therefore, the method reliably detects differences in the number of infected cells and provides estimates comparable to culture-based methods.

### Single-cell based quantification of viral infection in the environment

We adapted the iPolony method to meet the challenges of the complexity of natural communities. The initial steps are similar to those described above for model systems. First, infection in field samples is halted by fixation and flash freezing, enabling simple and rapid sample collection in the field. Samples can be stored for at least a year without affecting infection levels allowing flexible analysis time (Supplementary text, Supplementary Fig. 1). Second, cell sorting with a flow cytometer removes cells from complex communities, from free viruses, and from fixative-containing seawater that inhibits the polony reaction [33]. Furthermore, with our cytometer configuration sorting concentrates cells of interest to ~1000 ± 200 cells µl^−1^, which enables reactions to simultaneously screen up to ~10,000 cells for viral infection. Gels with fewer than 5–10 polonies per slide are considered to be below the limit of accurate quantification [33]. Thus, the method has a sensitive detection limit of 0.05% infection in a reaction with 10,000 cells per gel. Third, infection values are corrected for the presence of free viruses either by empirically testing the filtrate of sorted cells for free viruses, as was done for this study, or through a correction factor employed when free virus abundances are above an empirically determined threshold of when co-sorting with cells occurs (Supplementary results and discussion, Supplementary Fig. 4). Finally, differences in detection efficiency before, during and after genome replication in the infection cycle (Fig. 2) are used to adjust and provide bounds for infection estimates (see Supplementary text) since microbial populations in the environment are likely at different infection stages (see below). The maximal difference in infection was 3.4-fold between the two most extreme scenarios where all infected cells were either prior to or after virus genome replication for both T4-like and T7-like cyanophage infection assays, respectively. Therefore, using latent period weighted efficiencies to calculate infection values in natural samples provides accuracy within a ~3-fold range. These procedures allow for sensitive and high-throughput detection of viral infection from environmental samples.

### Low extent of viral infection of *Prochlorococcus* in the North Pacific Subtropical Gyre

Next we assessed whether viral infection exerted a significant control on *Prochlorococcus* abundances. We used the iPolony method to measure instantaneous levels of infection of *Prochlorococcus* at the high temporal resolution of every 4 h over 7 diel periods in the surface mixed layer in the North Pacific Subtropical Gyre (NPSG) in the summer of 2015. A Lagrangian sampling strategy was implemented to sample the same water mass during the sampling period by following drifters centered at 15-m depth [40].

**Fig. 4:**
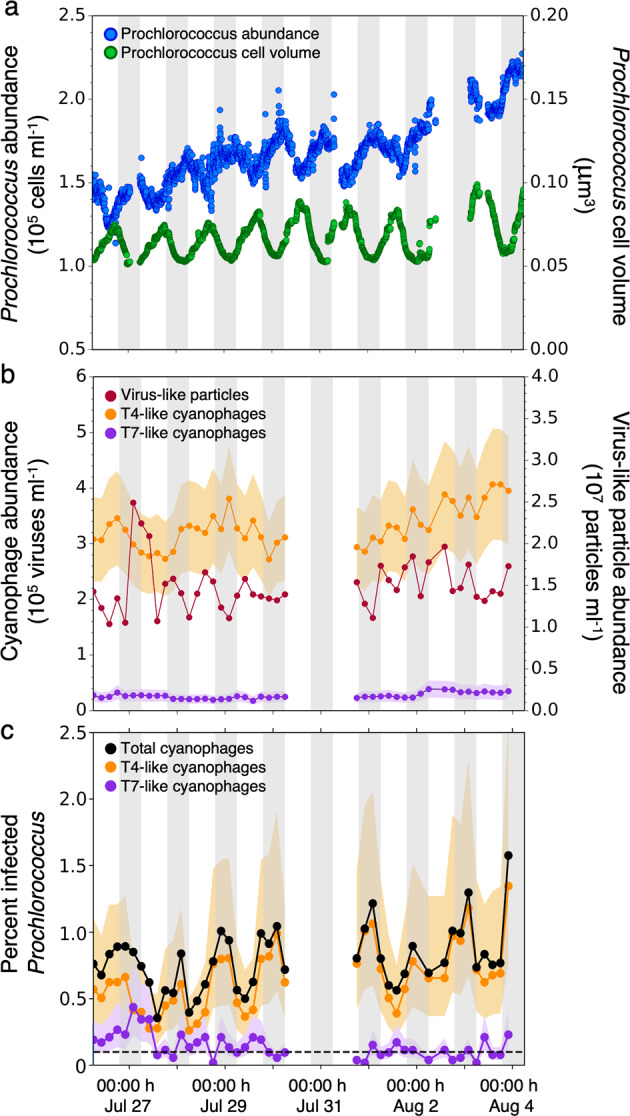
Diel dynamics of *Prochlorococcus* and cyanophages in the North Pacific Subtropical Gyre in 2015. Shaded regions indicate nighttime hours. **a** Abundances (blue) and cell volume (green) of *Prochlorococcus* following a Lagrangian water mass in the upper mixed layer. **b** Abundances of virus-like particles (red), T4-like (orange), and T7-like (purple) cyanophages. Shaded regions indicate the 95% confidence intervals of cyanophage abundance measurements. **c** Percent of virally infected *Prochlorococcus* by T4-like cyanomyoviruses (orange), T7-like cyanopodoviruses (purple), and total cyanophage (black) determined with the iPolony method. Shaded regions indicate bounds of infection assuming infection was entirely synchronous, and cells were either in the late stages of infection (lower bound) or in the early stages of infection (upper bound). Dashed lines indicate the limits of accurate detection for cyanophage infection. *Prochlorococcus* cell size, abundance and cyanophage infection all had statistically significant diel periodicity (RAIN rhythmicity test, *p*-values < 0.001), whereas VLP, T7-like, and T4-like cyanophage abundances were not periodic (RAIN rhythmicity test, *p*-value = 0.62, 0.42, 0.40 for VLP, T7-like, and T4-like cyanophage, respectively).

Picocyanobacteria abundances were measured continuously with an underway flow cytometer [38] and varied between 1.2–2.3 × 10^5^ and 0.4–1.6 × 10^3^ cells ml^−1^ for *Prochlorococcus* and *Synechococcus*, respectively (Fig. 4a, Supplementary Fig. 5). Because of the low abundances of *Synechococcus*, we focused our attention on *Prochlorococcus* as the most abundant primary producer in these waters. Free cyanophages were measured using the polony method [33, 34] and were found to be 2–4-fold more abundant than *Prochlorococcus* in the surface mixed layer (Fig. 4b). T4-like cyanophages ranged between 2.7 and 4.1 × 10^5^ viruses ml^−1^, and were an order of magnitude more abundant than T7-like cyanophages that numbered 1.7–3.8 × 10^4^ viruses ml^−1^. T4-like and T7-like cyanophages constituted ~2.2% and 0.1% of total VLPs, respectively. These abundances are within the range of our previously reported measurements for cyanophages in the upper mixed layer of the NPSG [34]. Furthermore, T4-like cyanophages, T7-like cyanophages, TIM5-like cyanophages, and cyanosiphoviruses were 89.0% (±1.2%), 3.55% (±0.96%), 0.35% (±9.5 × 10^−5^%), and 7.10% (±0.62%), respectively, of total cyanophage DNA sequence reads, normalized for genome size, in viromes taken in coordination with polony samples on the same cruise [46]. This provides independent evidence that T4-like cyanophages were considerably more abundant than T7-like cyanophages during this cruise and that other known cyanophage groups were not abundant [46].

Metagenomes and viromes were also used to assess the extent to which our degenerate primers and probes captured the major cyanophage types in these waters. The degenerate primers and probes which target the T4-like cyanophage *g20* gene captured the sequence variation in all 11 dominant T4-like cyanophage contigs [46] (Supplementary Fig. 6a). Sequence variation in the individual reads indicated that assemblies represented the dominant genotypes of cyanophages in the water and that primers and probes used in this study were compatible with the diversity of at least 93% of the individual reads (Supplementary Fig. 6b). Furthermore, sequenced amplicons from free cyanophages collected at 25- and 75-m depths during this cruise indicated that the primers captured a diverse set of T4-like cyanophage from within the population [34]. Therefore, any underestimation for cyanophage abundances and infection is likely to be minor and within the threshold of detection, unless due to a presently unknown, nonetheless abundant, cyanophage genotype.

Although cyanophages were abundant in the water column, only a small percentage of the total *Prochlorococcus* population were infected in all samples analyzed over all 7 days (Fig. 4c). Infection averaged 0.79% of the total *Prochlorococcus* community spanning from 0.35 to 1.6% or 520 to 3100 virally infected cells ml^−1^ (Supplementary Fig. 7). T4-like cyanophages accounted for over 80% of the observed infections. Infection by T7-like cyanophages was at or below the limit of accurate quantification (<0.1% of the *Prochlorococcus* population after adjusting for cyanophage detection efficiency) for the majority of the sampling period (Fig. 4c). These data highlight the importance of understanding the nature of specific virus–host interactions as different virus lineages have significantly different impacts on cyanobacterial communities.

Our observation that 0.35–1.6% of *Prochlorococcus* is infected, with estimates of 0.35–4.8% mortality (see below), is striking given the current paradigm that 10–40% of all cells are lysed by viruses each day [10, 11, 13–15, 36, 47, 48]. How can our observations be reconciled with these previous studies? Viruses were estimated to contribute between −21 and 22% of *Prochlorococcus* mortality in oligotrophic surface waters using the dilution method [25], which indirectly assesses mortality and lacks the sensitivity to accurately measure low infection levels [27, 49, 50]. A wide range of mortality values from <0.1 to 46% have also been reported using contact rates [18, 19] or production-decay calculations [24] as a proxy for infection. These approaches require enumeration of cyanophages and have employed methods that systematically underestimate abundances [11, 33]. They also rely on assumptions that are poorly constrained for environmental populations including variable burst sizes and decay rates as well as high virus infectivity and host susceptibility, which introduce inaccuracy and lead to significant overestimations of mortality. For example, estimated infection frequencies would be lower by up to 10-fold if decay rates based on longer reported estimates [51] were used. Finally, the frequency of visibly infected cells method identifies cells containing viral particles (capsids) and uses debatable conversion factors for the period of time that particles are visible to estimate mortality [16, 52, 53]. For example, 30% of picocyanobacteria mortality was attributed to viruses assuming capsids are visible for 10% of the latent period based on a heterotrophic bacteriophage system [16], whereas had 50–60% been used based on cyanophage systems 5–6% mortality would have resulted given the same data [18]. The iPolony approach improves on these most commonly used methods by directly measuring infection of tens of thousands of cells at all stages of the infection cycle and uses fewer and more well-constrained assumptions in converting infection to mortality estimates. This results in higher sensitivity with improved precision, reducing methodological variability and enabling a more accurate evaluation of viral mortality in natural systems.

Nonetheless, as a means to independently verify our estimates of 0.35–1.6% infection, we enumerated the number of single-cell genomes of *Prochlorococcus* collected from oligotrophic surface waters [54] that contain cyanophage contigs. While no single sample had enough cells sequenced to adequately determine infection, out of 298 sequenced *Prochlorococcus* cells from many geographically separate regions, 3 had cyanophage contigs in their genome assemblies [54], equivalent to ~1.0% infection. Thus, while not a robust means of quantification at low levels of infection, single-cell genomics provided independent support for our findings with the iPolony method.

### Diel periodicity of infection

*Prochlorococcus* abundances displayed diel periodicity (*p*-value <0.001), with numbers increasing at night and decreasing during the day (Fig. 4a). Changes in *Prochlorococcus* cell size were also periodic (*p* < 0.001) with cell volume decreasing by nearly half during nighttime due to cell division and respiration (Fig. 4a). This suggests that cells were dividing almost once a day consistent with previous observations in the region [5, 7]. A daily doubling of the entire *Prochlorococcus* population would be expected to produce ~1–2 × 10^5^ cells ml^−1^ each night if there was no mortality. However, the *Prochlorococcus* population only increased by 0.2–0.5 × 10^5^ cells ml^−1^ after dusk indicating tightly coupled loss factors that maintained relatively constant *Prochlorococcus* abundances. Despite the diel periodicity in *Prochlorococcus* populations, standing stock abundances of VLPs and cyanophages in the water column showed no diel periodicity (Fig. 4b) (*p*-value = 0.62 for VLPs, *p*-value = 0.40 for T4-like cyanophage, *p*-value = 0.42 for T7-like cyanophage).

In contrast to cyanophage abundances, viral infection of *Prochlorococcus* displayed diel periodicity (Fig. 4c) (*p*-value <0.001). Viral infection was relatively stable and low during daylight hours (0600–1400 h) and increased ~2-fold toward dusk (1800 h), which coincided with the peak in *Prochlorococcus* cell division (Fig. 4a, c). At night (1800–0200 h) infected cell abundances were approximately two times greater compared to daylight hours (0600–1400 h) with maximal infection detected between 2200 and 0200 h. The observed increase in percent infection at night may be due to increased infection, increased detection of infection because infection had progressed into stages of phage genome replication, decreased lysis, or a combination of these factors. Finally, infected cell abundances dropped ~2-fold between 0200 and 0600 h indicating that most infected cells underwent an early morning lysis event. This punctuated lysis may be an important event for the surrounding heterotrophic bacterial community which are likely to be utilizing the cyanobacterial lysate and may be partially driving the observed diel patterns in heterotroph metabolism [55]. It is important to note that a fraction of *Prochlorococcus* cells was always infected by T4-like cyanophages with between 450 and 1400 infected cells ml^−1^ found in morning hours (Supplementary Fig. 7). Thus, infection appeared to be strongly, but not entirely, phased to the light-dark cycle as there was some degree of asynchronous infection.

There is a clear decoupling between the low and cyclical infection dynamics and high and relatively invariable cyanophage abundances. We hypothesize that relatively slow cyanophage particle decay rates allow for the accumulation of a high standing stock of viruses which obscured the small periodic signal in daily changes in cyanophage abundances. We estimate that the turnover of the T4-like cyanophage standing stock of 3.3 × 10^5^ phages ml^−1^ would occur every 8–23 days assuming an average burst size of 12 T4-like cyanophages cell^−1^ (Supplementary Table 4), an average of 1200 *Prochlorococcus* cells ml^−1^ are lysed by T4-like cyanophages during each infection cycle (Supplementary Fig. 7), and 1–3 infection cycles occur each day (see below). This viral turnover duration is at the slower end of a wide range of previously reported decay rates for cyanophages, ranging from undetectable in dark conditions to between 0.048 and 2.01 d^−1^ in surface waters (equivalent to turnover times of 0.5–21 days) [17, 51]. Furthermore, decay rates are typically based on the decline in infective titers [17, 19, 51]. We expect particle decay to be slower as it not only requires a loss of infectivity but also degradation of gDNA.

Transcriptional activity of cyanophages conducted during the same cruise from the same depth [46] provide qualitative support for the phasing of infection. Cyanophage transcriptional activity peaks in the late afternoon and is at a minimum at dawn, which is also consistent with other reports of cyanophage transcription in the NPSG [56, 57]. Notably, cyanophage DNA and RNA metabolism genes are maximally transcribed at dusk [57], suggesting that viruses are replicating their genomes during or just after the time of host cell division [56]. Irrespective of viral infection, *Prochlorococcus* maximally expresses ribonucleotide reductases just prior to cell division [58]. This creates a large pool of free nucleotides for host cell division that could be efficiently utilized by an infecting virus for its own genome synthesis. We hypothesize that the consistent synchrony of *Prochlorococcus’* cell cycle to the light/dark cycle creates differences in the availability of intracellular resources throughout the day and may have selected for the phasing of cyanophage genome replication to coincide with host genome replication in order to maximize virus production.

The nighttime increase in cyanophage infection was unexpected as different lines of evidence suggest that darkness can prevent or reduce phage adsorption, reduce burst sizes, and lengthen or halt the infection cycle in laboratory experiments [59–62]. Furthermore, cyanophage genes are transcribed less [61] and cyanophage have decreased genomic DNA replication [35, 59, 61, 63] in the dark. However, the effects of darkness differ for different cyanophages even in a single family of T4-like cyanophages [62]. Thus, various cyanophages in nature are also likely to respond differently to darkness, with some capable of adsorption at night, some capable of genome replication and some capable of neither. The decrease in cyanophage transcription observed near dawn is likely to be at least partially due to a 2-fold drop in infected cell abundances. This significant drop in infection prior to sunrise suggests that there may be a presently unknown mechanism that induces the early morning lysis event.

### Estimating daily mortality and frequency of encounters that result in infection

We used the high frequency instantaneous measurements of viral infection to estimate the daily contribution of viruses to the mortality of *Prochlorococcus* (Supplementary text). Mortality is caused by lysis and is therefore relative to the number of infection cycles that can be completed in the population turnover time, which is approximately a day for *Prochlorococcus* in oligotrophic surface waters [5, 7]. Thus, the bounds of daily *Prochlorococcus* mortality fell between 0.35% (using the lower limit of instantaneous infection of 0.35% and 1 infection cycle a day) and 4.8% (using the upper limit of instantaneous infection of 1.6% and 3 infection cycles per day) (Fig. 4c). These bounds are based on the average latent period for numerous T4-like cyanophages (7.9 h, *n* = 9, Supplementary Table 4) determined in culture and assume all infections lead to lysis, which may be complicated by cells which overcome infections via intracellular resistance [39]. Over the course of the last 3 days of the sampling period, T4-like cyanophages increased by 76,000 cyanophages ml^−1^ whereas T4-like infected cells increased by 500 cells ml^−1^. Had there been a single infection cycle per day, a burst size of ~150 T4-like viruses per infected cell would be required to produce 76,000 T4-like cyanophages, which is 3 to 13-fold greater than measured burst sizes of 12 and 20–50 viruses cell^−1^ for infection of *Prochlorococcus* and *Synechococcus*, respectively, under optimal culture conditions (Supplementary Table 4). This suggests that multiple infection cycles occurred each day and that different subsets of the *Prochlorococcus* population would be infected at different times over the diel cycle. Our observation that infection was always detected and not completely synchronized supports this hypothesis (see Fig. 4).

The estimated daily mortality values are surprisingly low considering the high abundances of *Prochlorococcus* and free cyanophages. In order to compare the number of expected encounters to the number of empirically observed infections, we used encounter rate theory [64], which does not consider viral infectivity and host susceptibility, to estimate the number of potential interactions between *Prochlorococcus* and T4-like cyanophages based on diffusion alone. An individual *Prochlorococcus* cell should encounter one cyanophage every 54 h for a total of 70,000 encounters per day in a population of 1.6 × 10^5^ *Prochlorococcus* cells ml^−1^, which is the theoretical maximum rate given the average cell and virus concentrations observed at this time. If all encounters were to result in infection and lysis, viruses would be expected to kill 44% of *Prochlorococcus* daily. Yet our estimates of 0.35–4.8% daily mortality suggest that only 0.80–11% of the encounters resulted in infection.

Multiple mechanisms may explain the difference in predicted mortality based on encounter rates and the estimates of mortality, including the loss of infectivity, low adsorption efficiency, and host resistance. If each mechanism was solely responsible for mitigating infectious encounters, it would require that ~4.2% of cyanophages would be infectious, only 4.3% of encounters would result in adsorption, or 96% of *Prochlorococcus* (1.53 × 10^5^ cells ml^−1^ out of 1.6 × 10^5^ cells ml^−1^) would be resistant to their co-occurring viruses, to reconcile the observed infection rate with that expected from encounter theory. Instead, it is likely that all three mechanisms contribute to the observed infection frequencies. Previous reports in surface waters indicate that 20–92% of newly produced cyanophage lose infectivity per day [17, 65]. In cultured host-virus systems, 5–25% of encounters between infective phages and sensitive hosts result in adsorption and infection [66, 67]. While it is not known what fraction of wild *Prochlorococcus* populations is susceptible to any given virus, *Prochlorococcus* populations consist of hundreds of distinct types [68] with different sensitivities to co-occurring phages [69, 70]. Therefore, we suggest that a combination of these mechanisms, balanced at 10–35%, mitigates infections and thus sustains coexisting cyanobacteria and virus populations despite high *Prochlorococcus* cyanophage encounter rates (see Supplementary discussion, Supplementary Fig. 8).

## Conclusion

We have developed a single-cell method for viral infection—‘iPolony’—that enables the direct, sensitive quantification of infection in natural microbial populations and provides a high-throughput means to assess infection by different virus groups for specific host taxa. Using this method, we found low viral infection of *Prochlorococcus* (~1%) in contrast to measurements of high cyanophage abundances in the same body of water. These joint measurements indicate that high free virus abundances do not necessarily equate with high levels of infection and suggest that cyanophages were responsible for ~0.35–4.8% of daily mortality. Our finding is in contrast to the foundational tenet and generally held assumption, based on observations of high abundances of viruses in aquatic systems, that make a direct link between high free virus abundances and high microbial mortality [10, 11, 13, 15, 17–19, 23, 24, 71]. Instead, we have shown that high virus abundances should not be used as proxies for high levels of infection.

Given our estimate that virus-mediated mortality is relatively small, we hypothesize that other factors were likely to have been responsible for the majority of *Prochlorococcus* death in the surface waters of the North Pacific Subtropical Gyre at the time of sampling. Grazing is an obvious candidate and removal of the majority of the picocyanobacterial production (6.7–13.3 × 10^4^ *Prochlorococcus* cells mL^−1^ d^−1^ removed) was estimated to support the nanoflagellate population size and growth rates [72]. The combined virus- and nanoflagellate-mediated mortality could account for total mortality needed to balance the estimated division rates. However, other sources of *Prochlorococcus* death that have yet to be identified and quantified may also contribute.

Different sources of mortality are expected to differentially impact the flow of carbon and energy through the open ocean ecosystem. A grazing dominated system is hypothesized to transfer organic matter and energy produced by primary producers to higher trophic levels. In comparison, in a viral lysis dominated system organic matter would be shunted to the heterotrophic bacterial community where it is remineralized [10, 12]. Thus, measurements of viral infection using the iPolony method for key microbial taxa, like *Prochlorococcus*, can help illuminate the pathways and extent to which viruses influence the greater microbial community and biogeochemical cycles. We speculate that the major impact of viruses on *Prochlorococcus* in oligotrophic surface waters like the NPSG may reside in catalyzing the diversification of host populations [68–70].

Moving forward, the application of this method will enable mapping of cyanophage–cyanobacterial dynamics at high spatial and temporal resolution for multiple oceanic regions, sites, and depths to determine the natural variability of viral infection between environments and evaluation of the importance of viruses in controlling cyanobacterial populations. Such data can then be used to model ecosystem wide impacts of cyanophage infection in the oceans, including the effect on host population dynamics and the fate of autotrophically derived organic matter as it moves through the ocean ecosystem. The further adaptation of the method for other major virus families and microbial hosts, both heterotrophs and autotrophs, provides the prospect to evaluate the extent to which viruses control other microbial taxa and to expand such models to major constituents of ocean ecosystems.

## Supplementary information

Supplementary Information
