# Supplementary material for: A single-cell polony method reveals low levels of infected Prochlorococcus in oligotrophic waters despite high cyanophage abundances
Source: ISME J. 2020 Sep 11;15(1):41–54. doi: 10.1038/s41396-020-00752-6 (PMC7853090; doi:10.1038/s41396-020-00752-6)
Supplement: Supplementary file 1 — Supplementary Information [file 41396_2020_752_MOESM1_ESM.pdf]

Supplementary Information

Supplementary results and discussion 1-4

Methods development: Cell permeabilization, Single virus genome detection inside cells, Efficiency of detection during the latent period, Co-sorted virus contamination, Sample storage conditions

Balancing parameters that mitigate infectious encounters

Supplementary methods 4-8

Cyanobacterial growth and infection conditions

Phage lysate preparation

rbcL primer and probe design

Single virus genome detection inside cells

Comparison of metagenomes and polonies

Ecological model of viral infection

Supplementary tables 9-12

Supplementary Table 1: Methods for assessing viral infection in the environment

Supplementary Table 2: Primers and probes for standard PCR, qPCR, and polony reactions

Supplementary Table 3: The MOIs used in infection experiments and the expected % of infection according to a Poisson distribution

Supplementary Table 4: Infection characteristics of T4-like and T7-like cyanophages from literature

Supplementary Table 5: Sample locations and analyses

Supplementary figures and figure legends 1-8 13-20

Supplementary Figure 1: Storage effects on measurement of infected cells

Supplementary Figure 2: Storage effects on sorted cells

Supplementary Figure 3: Lysozyme treatment does not improve cell permeabilization

Supplementary Figure 4: Correction for co-sorted free viruses

Supplementary Figure 5: Abundances of Synechococcus *over the sampling period*

Supplementary Figure 6: Comparison of environmental T4-like cyanophage g20 *sequences to degenerate primers and probes*

Supplementary Figure 7: Diel periodicity in infected Prochlorococcus *cell abundances*

Supplementary Figure 8: Solution space for the frequencies of mechanisms that mitigate infection

References for supporting information 21-23

Supplementary results and discussion

Methods development

Cell permeabilization

We tested several methods to efficiently and quantitatively lyse cells after sorting to ensure maximal detection of infected cells. Performing in-gel heat lysis for 15 min at 94 ºC showed that it was effective at lysing both *Prochlorococcus* and *Synechococcus* cells since an average of 86% of infected cells were detected by the iPolony method in phage growth experiments (Fig. 2). We explored whether including a lysozyme treatment step in addition to heat lysis would further increase permeabilization efficiency. For this purpose, the polony procedure was adapted for detection of *Synechococcus* genomic DNA using the *rbcL* gene (Supplementary methods). *Synechococcus* sp. strain WH8109 cells were used as template for optimization assays. Incorporating the lysozyme treatment (ranging from 0.5 mg ml^{-1} to 10 mg ml^{-1} lysozyme) in-gel either before the diffusion step or with the diffusion mix, was not possible because it either inhibited or caused low efficiency of polony formation, respectively. The incubation of cells with 10 mg ml^{-1} lysozyme for 1 h at 37 ºC in-solution (73) prior to addition to the gel mix did not inhibit the polony reaction. Therefore, cell permeabilization with lysozyme was tested in buffers with either 0.75 M sucrose solution (74) or differing percent of artificial seawater medium (25%, 50%, 100% ASW). However, no increase in permeabilization efficiency was observed compared to treatments without lysozyme (two-tailed paired t-test, $p=0.56$) (Supplementary Fig. 3). Thus, heat lysis alone was used to permeabilize cells.

Single virus genome detection inside cells

The above results with the *rbcL* gene show that iPolony can detect single gene copies in *Synechococcus* cells. However, *Synechococcus* WH8109 is polyploid with 4 genome copies present at any one time (39). Therefore, to verify that single virus genes can be detected by iPolony, we transformed *E. coli* with a plasmid containing a single copy of the Syn9 phage *g20* gene and expressing YFP to enable fluorescence-based flow cytometric sorting. Three cultures of the strain were starved for 3 hours to prevent plasmid DNA replication (75) in the presence of kanamycin to prevent plasmid loss. The number of plasmid copies $cell^{-1}$ was determined by qPCR for the Syn9 *g20* gene with specific primers and an average of 1.01 ± 0.29 *g20* copies $cell^{-1}$ was found (n=3). The iPolony method with degenerate *g20* primers and probes detected 10% of the *E. coli* cells with the phage *g20* gene (n=3). This indicates that single copy genes are detected by the iPolony method, but at lower frequencies than free T4-like cyanophages (34).

Efficiency of detection during the latent period

The detection efficiency of infected cells changes over the latent period which is related to the number of viral genome copies in the cell (Fig. 2). Detection efficiencies at different stages of the infection cycle were assessed for 3 virus-host pairs: cyanophage Syn5 on *Synechococcus* sp. strain WH8109 (Fig 2. a, c, e), S-TIP37 on the same *Synechococcus* host (data not shown), and S-TIM4 infecting *Prochlorococcus* sp. strain MIT9515 (Fig. 2 b, d, f). All cyanophage growth experiments were conducted at an MOI of 3. Based on these experiments, three bins with different efficiencies were created to represent the time prior to, during, and after genome replication. The first bin was assigned an efficiency of 25%, the average of detection efficiencies of the three virus-host pairs, S-TIM4, S-TIP37, and Syn5, with efficiencies of 11%, 20%, 43% respectively. The second detection efficiency bin was assigned 55%, the average between the first and third bins. The third bin has an efficiency of 86% based on the maximal detection

efficiency observed in the S-TIM4 and Syn5 growth experiments (Fig. 2). Cyanophage growth experiments with T7-like (n=3) and T4-like (n=9) cyanophages (Supplementary Table 4) indicated that, on average, single virus genome copies exist prior to DNA replication for 23% and 30% of the latent period, that genome replication occurs for 27% and 35% of the latent period, and 50% and 35% of the latent period is after genome replication in T4-like and T7-like cyanophages, respectively. Therefore, the efficiencies of the three bins were weighted according to the average duration of each respective stage of infection for each phage family.

Co-sorted virus contamination

Potential co-sorting of free viruses can be a significant issue when sorting is based on autofluorescence properties of particles as cell sorters will not detect and abort events with non-fluorescent co-sorted viruses. Initial tests were performed by adding viruses to solutions of 1 μm yellow-green beads and sorting the bead-virus solutions. Either the T7-like cyanophage, P-SSP7, or the T4-like cyanophage, Syn9, were serially diluted 10-fold to achieve concentrations ranging from 10^3 to 10^8 viruses ml^{-1} and added to the beads. Polonies from free viruses were detected in sorted solutions when the added virus concentrations were 10^5 viruses ml^{-1} and above.

We empirically tested the frequency of co-sorted viruses in environmental samples that spanned a range of infection values, free virus abundances, depths, seasons, and ocean basins (n=19, see Supplementary Table 5). *Prochlorococcus* were sorted from these samples and an aliquot of the sorted cells was filtered through a 0.2 μm syringe top filter (Acrodisc HT Tuffryn membrane, Pall Corp.). Both cells and filtrate were analyzed with the same iPolony reaction conditions for the abundance of T4-like and T7-like polonies. The abundance of total polonies observed was compared to the abundance of polonies in the filtrate (Supplementary Fig. 4). Co-sorted viruses were compared to the abundances of cyanophages in the water column determined from these samples to estimate thresholds of co-sorted virus contamination (Supplementary Fig. 4c-d). When T7-like or T4-like free cyanophage in seawater samples was greater than 5×10^5 or 3×10^5 cyanophage ml^{-1}, respectively, co-sorted viruses contributed to a low but constant proportion of the infection signal (Supplementary Fig. 4c-d). Regressions between uncorrected percent infection and percent infection corrected for co-sorted free viruses indicated that co-sorted viruses contributed to 8.2% and 19.5% for T7-like cyanophage and T4-like cyanophage infection, respectively (Supplementary Fig. 4e-f). Thus, a correction factor (0.918 for T7-like cyanophage or 0.805 for T4-like cyanophage) is applied to adjust for free virus co-sorting when co-sorted virus concentrations were not empirically determined and *in situ* free virus abundances reach these thresholds.

Sample storage conditions

The effects of storage time and conditions on measured infected cell concentrations were tested for both initial sample collection and after sorting. Locations and dates of sample collection used in this section can be found in Supplementary Table 5. First, we tested whether freezing samples affected infected cell concentrations. Water was collected from the Mediterranean Sea off Haifa, Israel on September 11, 2018 and fixed with glutaraldehyde (0.125% final concentration). *Prochlorococcus* and *Synechococcus* were sorted from one aliquot that was not frozen. The other aliquots were frozen in liquid nitrogen and stored at -80 ºC for 5 hours, thawed, and then sorted. Sorted cyanobacteria from fresh and frozen samples were analyzed for the extent of viral infection by T7-like and T4-like cyanophages (Supplementary Fig. 1a). Freezing had no significant effect on percent infection compared to samples that were

not frozen (n=5, paired two-tailed t-test, p= 0.69). Thus, samples can be flash frozen without affecting the number of infected cyanobacteria measured.

To test the effect of long-term storage of samples at -80 ºC on infected cell abundances, samples were collected and stored in replicates. One set of samples collected from the Mediterranean Sea was analyzed for percent infection of *Prochlorococcus* and *Synechococcus* initially on the day of collection (unfrozen) and again 1 week, 6 weeks, and 5 months after collection (Supplementary Fig. 1b). To test storage over >5 months, a second set of samples (n=8) from the North Pacific Ocean were initially analyzed for *Prochlorococcus* percent infection 2 months after collection. Frozen replicates of these samples were then analyzed 1 year after the initial analysis (Supplementary Fig. 1b). There was no significant effect of storage time on the percent infection relative to the initial analysis (one way ANOVA, F(5,31)=0.64, p=0.67). These results were consistent across samples collected from different oceans that varied in their absolute infection percentage. Thus, virally infected cells are stable long-term when flash frozen in liquid nitrogen and stored at -80 ºC.

We tested the effects of different storage conditions on sorted cells in order to see if sorting and polony analysis could be performed on separate days due to the time intensive nature of performing sorting and the polony analysis on the same day. We tested this on *Prochlorococcus* cells as they are considered more sensitive than *Synechococcus*. First, *Prochlorococcus* cells were sorted from 3 environmental samples and were analyzed on the same day as they were sorted. An aliquot of sorted cells were also flash frozen in liquid nitrogen, stored at -80 ºC, and percent infection reanalyzed after 7 days. The number of polonies post-freezing decreased significantly (n=3, paired two-tailed t-test, p = 0.028) for *Prochlorococcus* cells infected by T4-like and T7-like cyanophage by an average of 47.5% and 58.7%, respectively (Supplementary Fig. 2a). Thus, *Prochlorococcus* cells cannot be frozen and stored after sorting without significantly affecting the number of infected cells measured. Second, we tested whether cells could be stored at 4 ºC without affecting infection levels. *Prochlorococcus* was sorted from two samples, and T4-like and T7-like cyanophage infection analyzed on the same day and 1, 2, 4, and 7 days post sorting after storage at 4 ºC in the dark (Supplementary Fig. 2b). Infection percentages decreased significantly over time (repeated measures ANOVA, F(4,12) = 6.119, p<0.05). In 3 of the 4 analyses, infection decreased 22-57% compared to samples analyzed on the same day (Supplementary Fig. 2b). Thus, these data indicate that sorted *Prochlorococcus* cells cannot be stored and polony analysis should be done on the same day as sorting.

<u>Balancing parameters that mitigate infectious encounters</u>

The graphical solution space in Supplementary Fig. 8 shows the range of parameters for loss of virus infectivity, inefficient adsorption to host cells, and reduced host susceptibility to infection which are needed to mitigate the high number of host-virus encounters based on encounter theory, which yields maximal encounter rates, to produce the empirically observed levels of infection. No a priori parameters were used to generate this solution space other than the average concentrations of *Prochlorococcus*, cyanophages, and percent infection. Via parsimony, we examine the range of values (the orange space in Supplementary Fig. 8) close to that in which all three parameters explain the gap in lysis rates from that expected by encounter theory. In this parameter regime, adsorption frequency, viral infectivity ranges, and host susceptibility all range between 10-35%.

This range of parameters falls within ranges of previously reported values. First, 5-25% is the median range of adsorption frequencies for ~70 host-virus pairs (66), consistent with our solution space for this parameter. Second, infectivity values can be derived from knowing the balance between cyanophage production and decay (17). Cyanophage decay ranges widely from 0.048 – 2.0 d^{-1} (19, 51). There are few data on cyanophage production, and this is typically derived from assuming that production balances decay (19). Between 20-90% of total viruses are thought to lose infectivity each day based on decay values (17, 65). We estimated that 14,440 T4-like cyanophages would be produced from the lysis of 1200 infected cells with a burst size of 12 viruses $cell^{-1}$ (see Results and Discussion), a conservative estimate based on 1 infection cycle occurring each day. The timescale of production balancing decay in this scenario is a 23-day turnover time. For 10-35% of cyanophages to be infective at steady state between ~28-99% of cyanophages would need to lose infectivity each day. This overlaps with the wide bounds of loss of infectivity each day noted above (17, 65).

Third, the fraction of susceptible hosts that exist in a given population is currently unknown. Hundreds of genomically distinct *Prochlorococcus* subpopulations coexist in a single water mass (68, 76). This could represent a lower bound if every subpopulation was infected by a distinct virus. However, it is well known that multiple cyanophages can infect a particular cyanobacterial isolate (18, 30, 77-79) and that a single phage can infect multiple cyanobacteria (18, 30, 39). Thus, in the field, a network of susceptible hosts and infective viruses is highly likely to be more complex with overlapping interaction networks (69, 80, 81). Thus, we cannot reliably evaluate the range of cyanobacterial susceptibility. It should be noted, however, that some estimates of susceptibility have been made for *Synechococcus* based on isolating cells and phages from a single water source and testing their cross-infectivity (18, 30) or based on contact-rate calculations (19). These calculations suggest that inshore *Synechococcus* are largely resistant (18, 19) while offshore *Synechococcus* are largely susceptible to co-occurring cyanophages (19).

Supplementary methods

Cyanobacterial growth and infection conditions

Cultures of *Synechococcus* sp. strain WH8109 and *Prochlorococcus* sp. strain MIT9515 were grown in artificial seawater medium (ASW) or Pro99 medium, respectively, at 22 ºC under a 14:10 h light-dark cycle at a light intensity of 7-10 µmol photons m^{-2} sec^{-2}. Cyanobacterial cultures were also grown on semi-solid media to produce a lawn of cells using the pour plating method (82). Briefly, *Synechococcus* or *Prochlorococcus* cells were mixed with either ASW or Pro99 medium containing Invitrogen Ultra-Pure low melting point agarose at a final concentration of 0.28% and poured into petri dishes. The helper bacterium *Alteromonas* sp. strain EZ55 was added to the cells to reduce oxidative stress and increase the efficiency of plating (83). The plated cultures were grown under the same conditions described above. Culture growth in liquid media was monitored via chlorophyll *a* fluorescence using a Turner 10AU fluorometer. Cell abundances were measured on a BD LSR II cytometer for *Synechococcus* or a BD Influx cytometer for *Prochlorococcus*.

Phage lysate preparation

Phage lysates of Syn5, S-TIP37, and S-TIM4 were prepared by infecting 25-30 ml volumes of cyanobacterial cultures with phages at a MOI of 0.1 to 0.6. After culture lysis, cell debris were precipitated by centrifugation at 8694xg for 15 min at 21 ºC and the supernatant

containing free phages was collected and filtered through a 0.2 μm pore size syringe filter (Acrodisc HT Tuffryn Membrane, Pall Corp.).

rbcL primer and probe design

The polony method for detection of cyanobacterial genomic DNA was used to assess the efficiency of cell permeabilization methods. Specific primers and probes were designed for the *rbcL* gene from *Synechococcus* sp. strain WH8109 (Supplementary Table 2). The primers were first tested by standard PCR and resulted in the amplification of the expected product with a size of 412 bp. The conditions for the polony method were optimized for these primers using extracted genomic DNA and were 1.5 μM acrydite-modified primer, 0.5 μM non-modified primer, 0.335 U/μl enzyme, 12.5% acrylamide gel, 50 ˚C annealing temperature, 35 PCR cycles, and 55 ºC probe hybridization temperature. These polony conditions yielded 31% ± 7% (n=4) efficiency of polony formation on extracted DNA.

Single genome copy detection in *E. coli*

The T4-like cyanophage Syn9 *g20* gene was cloned into the pMK-proCAT plasmid, a pUC57 derivative containing EcoRI and SpeI cleavage sites. Primers DR213F (GTCTAAGAATTCCACTAGATTCATCTAGAATCTTCTACATTGACGTTGGTAA) and DR213R (TCAGAAGTGCATCAAGGTCGAATACTTACCAACAAAAGGATCC) were used to amplify the *g20* fragment as well as to add EcoRI and XbaI sites at the 5' end and a SpeI site at the 3' end of the fragment. This 586 bp PCR fragment was then cloned into pMK-proCAT by EcoRI and SpeI cleavage and ligation. The resulting plasmid was named pG20-C1. Plasmid abundance was determined from the concentration of DNA based on absorbance at 260 nm and the molecular weight of the plasmid based on its sequence length.

A PCR fragment carrying single copy of the *g20* fragment, yellow fluorescent protein (YFP), and a kanamycin resistance gene was constructed using primers DR286F (TTAAGCACCGGAATTCCAC) and DR286R (CGAAAACTCACGTTAAGGG), and transformed into *E. coli* strain DH10b. To inhibit plasmid replication (75) and reach a single plasmid copy cell^{-1}, triplicate *E. coli* cultures grown in LB medium overnight at 37˚C with shaking at 250 rpm were starved by transfering to minimal M9 medium (Sigma) in the presence of kanamycin to retain plasmid selection. The LB medium was removed by two rounds of centrifugation and resuspension in M9 medium (Sigma) and a 50-fold dilution into M9 medium with kanamycin. The cultures were then incubated for an additional 3 hours at the original growth conditions. Cells (100 μL) were harvested, fixed with 0.1% glutaraldehyde, sorted based on YFP fluorescence and size and subjected to the iPolony protocol with *g20* T4-like degenerate primers and probes.

To confirm a single copy of the plasmid per cell, cells were collected on a 25 mm diameter, 0.2 μm pore-size polycarbonate membrane filter (Millipore, GVS). Intracellular DNA was extracted by a heat lysis method as described previously (84). The extracted DNA was used as a template for quantitative real-time PCR using primers Syn9_g20F/R (Supplementary Table 2), and the pG20-C1 plasmid (described above) was used to generate a standard curve for absolute copy number calculation.

Comparison of metagenomes to the polony method and polony primers

Virome sequence reads from the 2015 cruise (46) were downloaded from NCBI's Sequence Read Archive (Bioproject PRJNA358725). Sequences were quality trimmed to a Phred

score of 20 and quality controlled for a minimum length of 50 bp using BBDuk in BBtoolsv38.22 (sourceforge.net/projects/bbmap/). Quality controlled reads were than classified using Kaiju v1.7.0 (85) using the 'viruses 2019-02-05' database with default settings. All reads identified as cyanophage were summed by sample and taxonomic affiliation (T4-like cyanomyovirus, T7-like cyanopodovirus, TIM5-like cyanomyoviruses, and cyanosiphoviruses). T4-like myoviruses have larger genome sizes (~180kb)(86) than T7-like podoviruses (~45kb)(87) and cyanosiphoviruses (~30-110kb)(32) and thus may recruit more reads. Therefore, read abundances were normalized for genome size by dividing by a factor of 3.5, 1.5, and 1 for T4-like cyanophages, cyanosiphoviruses, and T7-like cyanophages. The normalized sum of each group was divided by the total number of cyanophage reads in each sample to calculate relative abundance of each phage group.

We checked whether the primers and probes used in the polony and iPolony methods to assess T4-like cyanophage abundance and infection captured the sequence diversity of the T4-like cyanophages in the environment. We downloaded the virus contigs assembled from the 2015 cruise (Bioproject PRJNA358725)(46) and assumed that assembled viruses were the major genotypes in the water at this time because they were abundant enough to generate long scaffolds and were reported to be high in relative abundance in the libraries (46). Genemark.hmm v3.25(88) was used to find open reading frames (ORFs) on scaffolds. ORFS were blasted against nr (downloaded February 21, 2019) to identify T4-like cyanophage *g20* sequences and checked against scaffold taxonomic assignments in Aylward et al. (46). The environmental T4-like cyanophage *g20* sequences and the Syn9 *g20* sequence as reference were aligned with MUSCLE v3.8.31(89)and visualized with AliView (90). We further assessed whether the sequence variation in the sequence reads was captured by the primers and probes. Sequence reads from the viromes used to generate the above assemblies were downloaded. A phylogenetic tree of *g20* nucleotide sequences was constructed based on the tree in Goldin et al. (34) using RAxML v8.1.20 (91). To obtain short reads, a database of all viromes was searched using blastn v 2.9.0 (92) at an e-value of $1x10^{-5}$ using representative cyanophage *g20* sequences as queries to broadly capture the diversity of *g20* sequences in the viromes. Short read sequences were aligned to full-length reference sequences using PaPaRa: Parsimony-based Phylogeny-Aware Read Alignment program 2.0 (93). Reads were placed on phylogenetic trees using the EPA: Evolutionary Placement Algorithm portion of RAxML v. 8.1.20. Reads that did not place within the T4-like cyanophage group were removed from the PaPaRa alignment. Alignments were trimmed to the primer and probe sequences used in the iPolony assays. Sequence logos were generated from these curated alignments using WebLogo v 2.8.2 (94).

<u>Ecological models</u>

Estimates of daily mortality

An ecological model containing susceptible *Prochlorococcus* cells, P, infected cells I, and viruses V, was created to infer the fraction of daily mortality that could be attributed to the viruses. Susceptible cells grow logistically with a per capita growth rate μ and carrying capacity K. Adsorption of viruses to cells occurs at a rate ϕ, and all other losses of susceptible cells are characterized by the rate m_1. Infected cells lyse following the latent period $L = 1/\lambda$ generating a burst size β of new virions. Other losses to infected cells are characterized by the rate m_2, which may differ from m_1. Finally, the virus decay rate is described by ω. This ecological system is described by the following differential equations:

$$\frac{dP}{dt} = \mu P\left(1 - \frac{P+I}{K}\right) - \phi PV - m_1 P$$
$$\frac{dI}{dt} = \phi PV - \lambda I - m_2 I$$
$$\frac{dV}{dt} = \beta(\lambda I) - \phi PV - \omega V$$

We assumed steady state, where *Prochlorococcus* mortality and growth are balanced, and the population growth is approximately μP^* assuming total cell density is much less than K. We note that the infected fraction f_i at steady state is $I^*/(P^*+I^*)$, which is approximately I^*/P^* given low rates of infection ($I^* << P^*$). The fraction of mortality attributed to viruses (f_{vm}) can then be written as the ratio of lysis to growth, i.e.:

$$f_{mv} = \frac{\lambda I^*}{\mu P^*} \approx \frac{\lambda}{\mu} f_I = \frac{T_p}{L} f_I$$

where T_p is the population turnover time and L is the infection latent period. Effectively, the fraction of virus-induced mortality is the number of infection cycles completed by the infected cell fraction within the population turnover time.

Estimating encounter rates

Encounter rates between *Prochlorococcus* and T4-like cyanophages were calculated using the Einstein equation for diffusion (95) and the Smoluchowski equation for particle encounters (64). Each particle is assumed to be a sphere and its movements governed by diffusion. The diffusion constant (D) for each sphere is:

$$D = \frac{k_B T}{6\pi\eta r}$$

where k_B is Boltzmann's constant ($1.38\text{x}10^{-8}$ µm^2 g s^{-2} K^{-1}) , T is the temperature of the seawater (296.15 K), η is the dynamic viscosity of seawater ($9.96\text{x}10^{-7}$ g µm^{-1} s^{-1}), and r is the radius of the sphere. Although T4-like viruses are not spherical and are composed of a 80-90 nm capsid and a 150 nm tail (96), we approximate their dimensions within the constraints of the model being a 0.15 µm diameter sphere. *Prochlorococcus* was assumed to have a 0.5 µm diameter. The encounter rate kernel (E) of one *Prochlorococcus* cell with cyanophages which is governed by diffusion is:

$$E = 4\pi(D_{Pro} + D_{virus})(r_{Pro} + r_{virus})$$

where r is the radius of a sphere and D is the diffusion constant of that sphere. The encounter rate kernel (E) was multiplied by the abundances of *Prochlorococcus* and the abundances of free cyanophages to calculate the total number of contacts between cyanophages and *Prochlorococcus* at the population level. The percent of encounters that resulted in infection was determined by dividing the total number of *Prochlorococcus* cells that were estimated to be killed per day, based on 1-3 latent periods derived from iPolony measurements (see above), by the estimated total number of encounters per day at the population level. Similarly, to estimate the percent of *Prochlorococcus* killed per day based on contact rates assuming 100% of

encounters resulted in infection, the total number of contacts d^{-1} ml^{-1} was divided by the abundances of *Prochlorococcus* ml^{-1}. We estimated the number of resistant cells assuming all viruses were infective and all contacts would lead to infection by taking the difference between the number of cells that encountered a virus based on contact rates and the number of cells killed based on polonies and then dividing this number by the total number of contacts d^{-1} ml^{-1}, or in other words, the number of cells that were contacted but not infected. It was assumed that no cell contacted the same virus twice. Thus, these represent maximum theoretical contact rates.

To visualize the solution space for virus infectivity, host susceptibility, and adsorption efficiency the encounter rate equation was adapted as:

$$C = zE*xV*yP$$

where E is the encounter kernel from the above equation, x is the infective fraction of the total virus population, V, (3.6×10^5 viruses ml^{-1}), y is the susceptible fraction of *Prochlorococcus*, P, (1.8×10^5 cells ml^{-1}), and z is the adsorption efficiency of a host-virus encounter. The function was parameterized as described above with the number of encounters that result in infection and lysis each day (C) being 0.79% as observed on average throughout the time series with the iPolony data. We assume that the system is in steady state where new encounters that lead to infection must balance the loss of infected cells each day.

Supplementary Table 1. Methods for assessing viral infection in the environment

Method type	Method	Measurement	Level of discrimination		Critical Assumptions	Throughput
			Virus	*Host*		
**Indirect - observational**	Contact rates (18, 24, this study)	Host and virus abundances (titers, qPCR, polonies)	None (titers), Specific for single virus genotypes (qPCR), virus families (polonies)	Depends on strain(s) of host culture used (titers), virus genotypes with known hosts (qPCR and polonies)	Theoretical adsorption rates, all cells susceptible to all viruses, all viruses are infective	10-20 samples: Hours (qPCR), Days-weeks (titers), 1-2 days (polonies)
**Indirect – experimental incubations**	Modified dilution incubations (26, 97)	Change in host growth rates after incubation with virus-free water	None	Flow cytometric populations	Dilution only affects mortality by viruses	1-10 experiments: 2-4 weeks
	Virus production incubations<br>• VLP production (20)<br>• Radiolabel incorporation (98)<br>• Fluorescently labeled virus tracers (99)	Change in VLP abundance after reducing background viruses or change in tracer or radiolabel levels over time	None	None	Virus burst size, steady-state virus production balances decay, radioactivity to virus production conversion factor	1-10 experiments:1-3 months
	Decay rates (17, 52)	Decay in viral titer or VLPs over time	None	Depends on strain(s) of host culture used for titers, none for VLPs	Steady-state virus production balances decay, virus burst size	1-10 experiments: Days-weeks (titers), 2-4 weeks (VLP)
**Direct-observational**	Visibly infected cells (16)	Frequency of cells harboring virus-like particles in electron micrographs	Morphological characterization	Morphological characterization	Length of time that capsids are visible during latent period	1-10 samples: 3-4 days
	Digital PCR (100)	Virus DNA detected inside spatially separated cells	Specific for single virus genotype	None	DNA inside cell is infection	20-50 samples: 1-2 days
	PhageFISH (101)	Cells with fluorescence of virus-specific probes	Specific for single virus genotype	Only for viruses with known hosts	DNA inside cell is infection	10-20 samples: 1 week
	Single cell genomics (102-104)	Presence of viral DNA within sorted cells	All virus types	All host types	DNA inside cell is infection	1-10 samples: Months
	iPolony method (this study)	Virus DNA detected inside spatially separated cells	Entire virus families	Flow cytometric populations	DNA inside cell is infection	10-20 samples: 1-2 days

Supplementary Table 2. Primers and probes for standard PCR, qPCR, and polony reactions.

Target phage/ cyanobacteria		Target gene	Primer/probe name	Sequence 5'-3'	Use/Reference
T7-like podophages	Degenerate primers	*DNApol*	Acr-534Rd	TGNWRYTCRTCRTGNAYRAA	Polony and iPolony (33)
			341Fd-15-NNN	NNNCCNAAYYTNGSNCAR	Polony and iPolony (33)
Clade A T7-like podophage	Degenerate probes		Cy5/Cy3-405AF(d+3i)	TAYTGYYTiATiTAYGGiGG	Polony and iPolony (33)
Clade B T7-like podophage			Cy5-405BF(d+3i)	TAYGCiTTYYTiTAYGGiGC	Polony and iPolony (33)
Subcluster B TIP42 podophage			Cy5-405BF(d+3i)_tip42	TAYTGiTTYYTiTAYGGiGG	
Syn5 podophage	Specific probe		CY5-1238F-Syn5	ATCGTAAGAGTGGCAAGGGAGTT	
	Specific primers	*HTTC*	Syn5_HTTC_RT_F	GTGATCTCTCCAGCCTCGAC	qPCR (33)
			Syn5_HTTC_RT_R	GGCTAACCATGAACACAACCTT	
T4-like myophage	Degenerate primers	*g20*	CPS1.2	ATHTTYTAYATHGAYGTNGG	Polony and iPolony (34)
			CPS8.2	ARTAYTTNCCNRYRWANGG	Polony and iPolony (34)
T4-like myophage	Degenerate probes		g20_cyano_env	RTCRTAYTGDATRTGITC	Polony and iPolony (34)
Non-cyano T4-like myophage			g20_env	GCRAARTRICCRTCYTK	Polony and iPolony (34)
Syn9 myophage	Specific primers	*g20*	Syn9_g20F	TCGTTTAGAAACAGAAACCACATTT	qPCR (this study)
			Syn9_g20R	AACTTTTGGAATTTAACTTCGTCAC	
Synechococcus strain WH8109	Specific primers	*rbcL*	Acr-8109rbcl708F	TCGCAGTCACGTTGAGGTAG	iPolony (this study)
			8109rbcl-1119R	TTCGAAGAGGGTTCCATCAC	iPolony (this study)
			708F-20	TCGCAGTCACGTTGAGGTAG	Standard PCR (this study)
			1119R-20	TTCGAAGAGGGTTCCATCAC	
	Specific probe		Cy5-rbcl-836R	GGTCTGGACTTCACCAAGGA	iPolony (this study)

Supplementary Table 3. The MOIs used in infection experiments and the expected % of infection according to Poisson distribution.

MOI	Expected % of infected cells
3	95
2	86.5
1.39	75
1	63.2
0.69	50
0.5	39.4
0.29	25
0.11	10

Supplementary Table 4. Infection characteristics of T4-like and T7-like cyanophages.

Virus family	Virus	Host strain	Burst size (viruses $cell^{-1}$)	Latent period (h)	Duration during latent period (h)			Reference
					Before replication	*DNA replication*	*After replication*	
T4-like	S-TIM4	*Prochlorococcus* MIT9515	n/a	8-10	1	2	5-7	This study
T4-like	P-TIM75	*Prochlorococcus* MED4	n/a	4	1	1	2	(39)
T4-like	P-HM2	*Prochlorococcus* MED4	12	8	0.5	2	5.5	(105)
T4-like	P-TIM68	*Prochlorococcus* MIT9515	12	12	4	2	6	(39, 106)
T4-like	P-TIM40	*Prochlorococcus* NATL2A	n/a	10	3	2	5	(39, 107)
T4-like	Syn9	*Synechococcus* WH7803, WH8102, WH8109	21-43	4	0.5	2.5	1	(37, 107)
T4-like	S-TIM4	*Synechococcus* WH8102	n/a	5	2	2	1	(39)
T4-like	S-PM2d	*Synechococcus* WH7803	45	12	3	4	5	(108, 109)
T4-like	S-CBM2	*Synechococcus* CB0101	28	15	n/a	n/a	n/a	(110)
T7-like	P-SSP7	*Prochlorococcus* MED4	n/a	8	2	2	4	(35)
T7-like	S-TIP37	*Synechococcus* WH8109	n/a	3	1.5	1.5	0	(36)
T7-like	Syn5	*Synechococcus* WH8109	n/a	1	0.1	0.7	0.2	This study

Supplementary Table 5. Sample locations and analyses for virus co-sorting and sample storage

Region	Depth (m)	Latitude	Longitude	Collection date	Co-sorted viruses	Fresh vs frozen	-80 °C storage	Freezing sorted cells	4 °C storage of sorted cells
Red Sea	0	29.46	34.94	Mar-19-2014	x				
Red Sea	20	29.46	34.94	Mar-19-2014	x				
Red Sea	40	29.46	34.94	Mar-19-2014	x				
Red Sea	60	29.46	34.94	Mar-19-2014	x				
Red Sea	80	29.46	34.94	Mar-19-2014	x				
Red Sea	100	29.46	34.94	Mar-19-2014	x				
Red Sea	140	29.46	34.94	Mar-19-2014	x				
Red Sea	0	29.46	34.94	Sep-16-2014	x				
Red Sea	20	29.46	34.94	Sep-16-2014	x				
Red Sea	40	29.46	34.94	Sep-16-2014	x				
Red Sea	60	29.46	34.94	Sep-16-2014	x				
Red Sea	80	29.46	34.94	Sep-16-2014	x				
Red Sea	100	29.46	34.94	Sep-16-2014	x				
Red Sea	140	29.46	34.94	Sep-16-2014	x				
North Pacific Ocean	5	23.49	-157.98	Apr-20-2016	x		x		
North Pacific Ocean	5	32.59	-158.00	Apr-24-2016				x	x
North Pacific Ocean	5	34.84	-158.01	Apr-25-2016	x		x	x	
North Pacific Ocean	5	35.68	-158.00	Apr-25-2016	x		x	x	
North Pacific Ocean	5	32.26	-158.01	May-31-2017	x		x		
North Pacific Ocean	5	33.45	-158.01	May-31-2017	x		x		
North Pacific Ocean	5	35.40	-157.98	Jun-7-2017	x		x		
North Pacific Ocean	5	34.50	-158.00	Jun-8-2017	x		x		
North Pacific Ocean	15	24.54	-156.50	Aug-2-2015	x		x		x
Mediterranean Sea	5	32.80	35.00	Sep-11-2018		x	x		

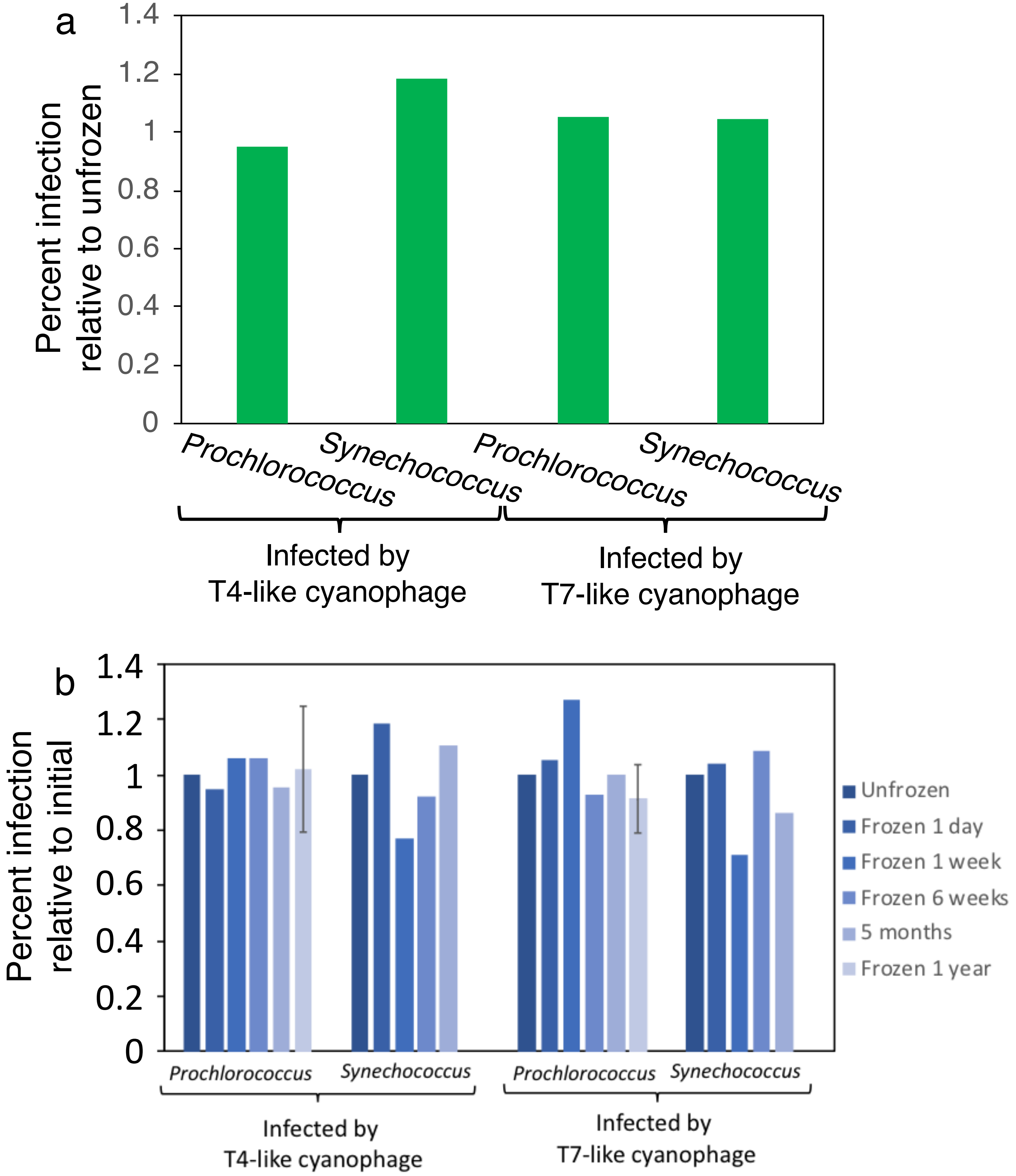

Supplementary Fig. 1. Storage effects on measurement of infected cells. **a,** The effect of freezing environmental samples on percent infection determined with the iPolony method. Replicate samples were collected, fixed, and either flash frozen and stored for 5 h at -80 °C or analyzed immediately without freezing. Bars represent the percent infection of *Prochlorococcus* and *Synechococcus* by T4-like or T7-like cyanophages in frozen samples relative to the percent infection assessed in unfrozen replicates. Freezing had no effect on percent infection (paired two-tailed t-test, p= 0.69). **b,** Long term storage of environmental samples at -80 °C. Replicate samples were analyzed for the extent of viral infection by T4-like and T7-like cyanophages after being stored for various lengths of time at -80 °C. The percent infection relative to the first analysis is shown. Decreasing shades of blue show the increasing length of time a sample was stored. Storage of at least one year did not affect infection values (one way ANOVA, F(5,31)=0.64, p=0.67).

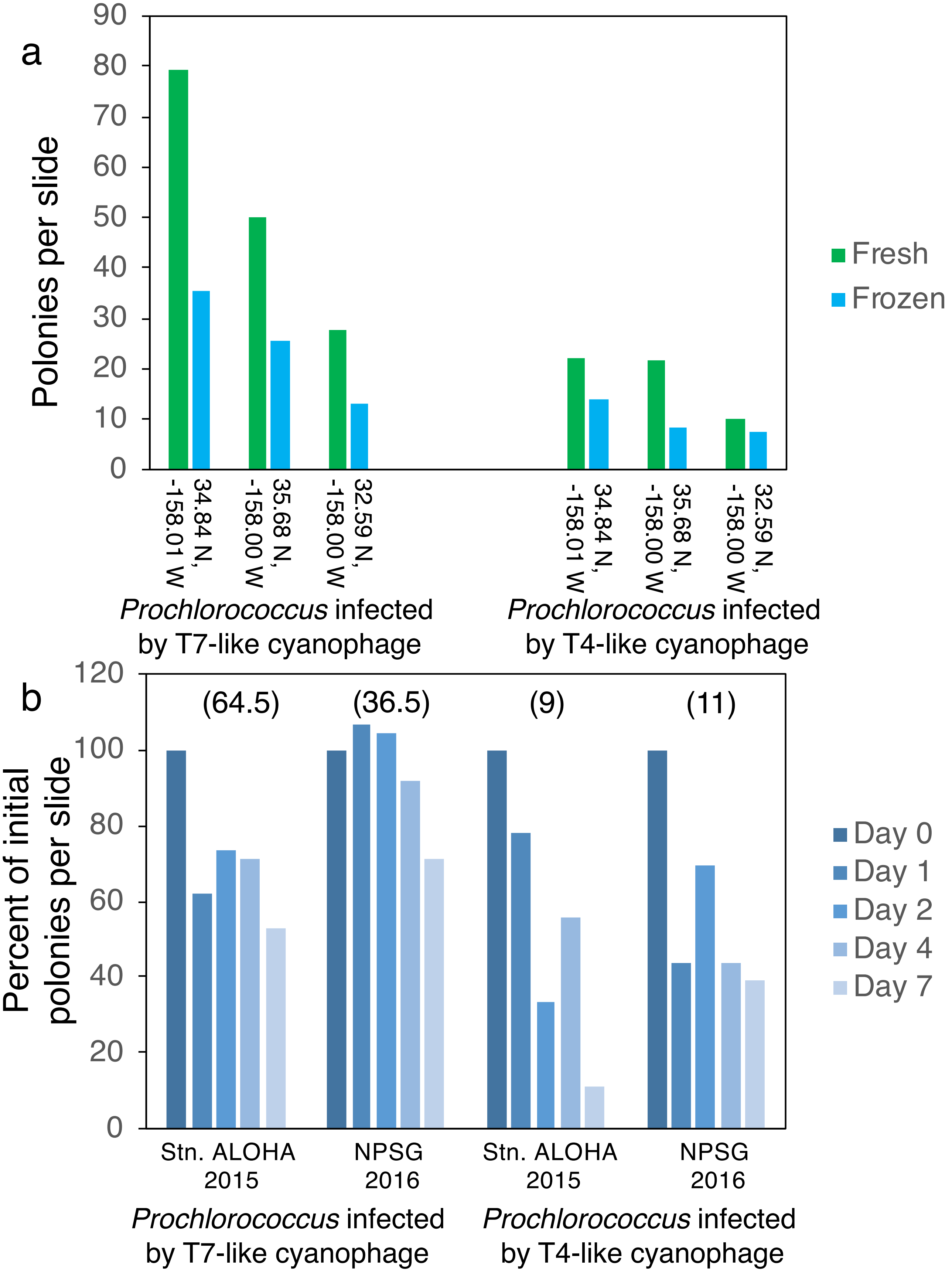

Supplementary Fig. 2. Storage effects on sorted cells. **a,** Storage of sorted cells at -80 °C. The number of T7-like (right) and T4-like (left) cyanophage polonies when sorted *Prochlorococcus* cells from three locations in the NPSG were analyzed initially on the day of sorting (green) or frozen in liquid nitrogen, stored at -80 °C, and analyzed 7 days later (blue). Freezing after sorting significantly decreased percent infection (paired two-tailed t-test, $p = 0.028$). **b,** Storage of sorted cells at 4 °C. *Prochlorococcus* was sorted and analyzed fresh or stored at 4 °C and analyzed after 1, 2, 4, and 7 days for viral infection by T4-like (right) and T7-like (left) cyanophages. The percent polony abundance is shown relative to the freshly analyzed sample. Initial total polony abundances are shown in parentheses above the bars. Percent infection decreased significantly over time when sorted cells were stored at 4 °C (repeated measures ANOVA, $F(4,12) = 6.119$, $p<0.05$).

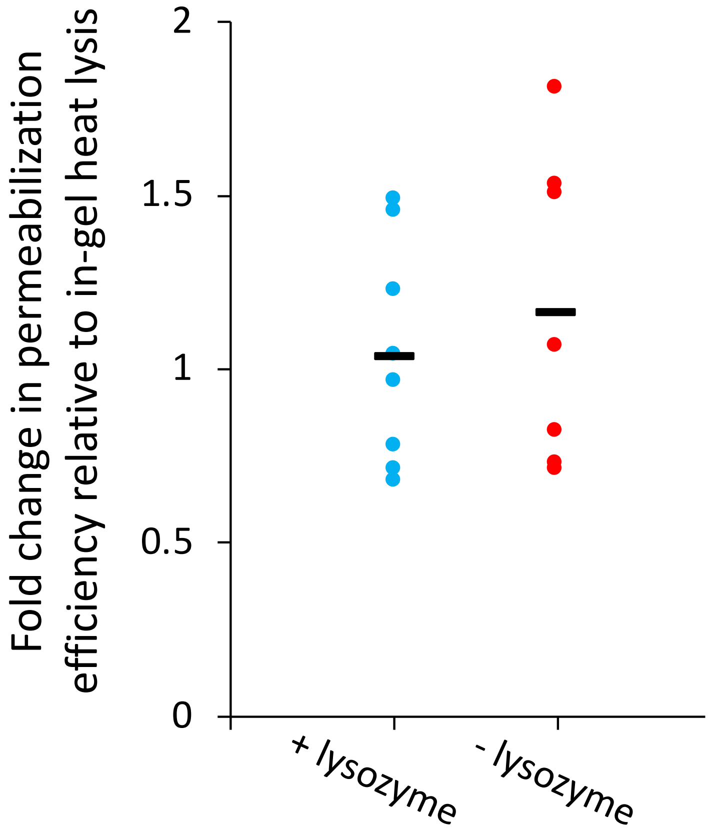

Supplementary Fig. 3. Lysozyme treatment does not improve cell permeabilization. The iPolony protocol for the *rbcL* gene was performed on *Synechococcus* sp. strain WH8109 cells treated with (blue) or without (red) lysozyme in different buffers for 1 h at 37 °C prior to in-gel heat lysis. The polony formation efficiencies for both treatments were normalized to the efficiency of controls where cells were treated with in-gel heat lysis alone. Addition of lysozyme did not affect polony formation efficiency beyond heat lysis alone (two-tailed paired t-test, $p=0.56$).

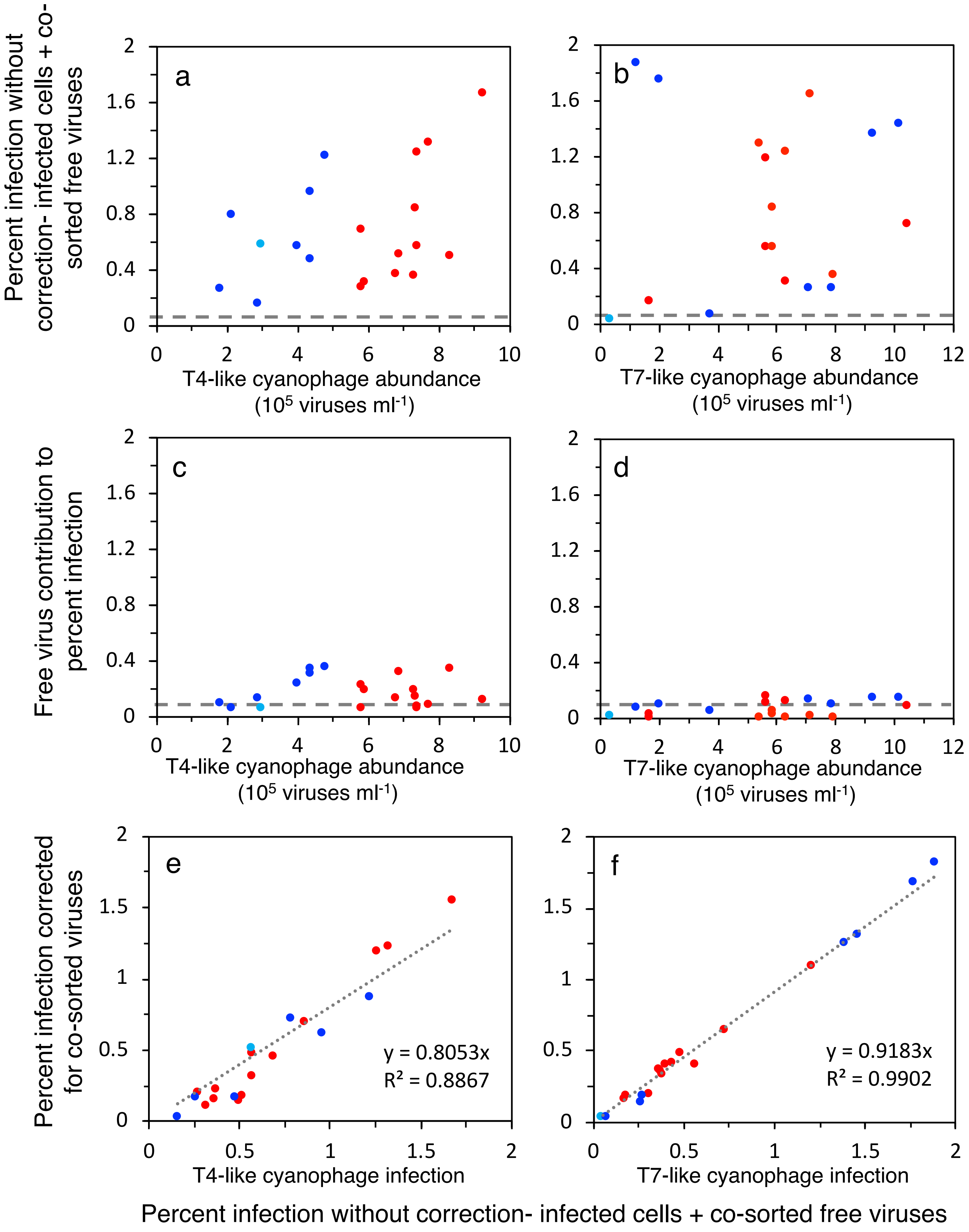

Supplementary Fig. 4. Correction for co-sorted free viruses. Samples were analyzed for the percent of infected *Prochlorococcus* by T4-like (**a,c,e**) and T7-like (**b,d,f**) cyanophages and for the abundance of co-sorted cyanophages in samples collected from the North Pacific Ocean (blue), Rea Sea (red), and during the 2015 diel sampling at station ALOHA in this study (light blue). Uncorrected percent infection (**a,b**) and the percent infection attributed to co-sorted viruses (**c,d**) are shown in relationship to cyanophage abundances in the water column. (**e,f**) Linear regressions between uncorrected and corrected percent infection values. The slope of the linear regression is used to correct infection values from environmental samples for which direct measurement of co-sorted free phage were not determined. Note that no correction was needed for this study as free viruses were below concentrations at which they were co-sorted with cells (**c,d**).

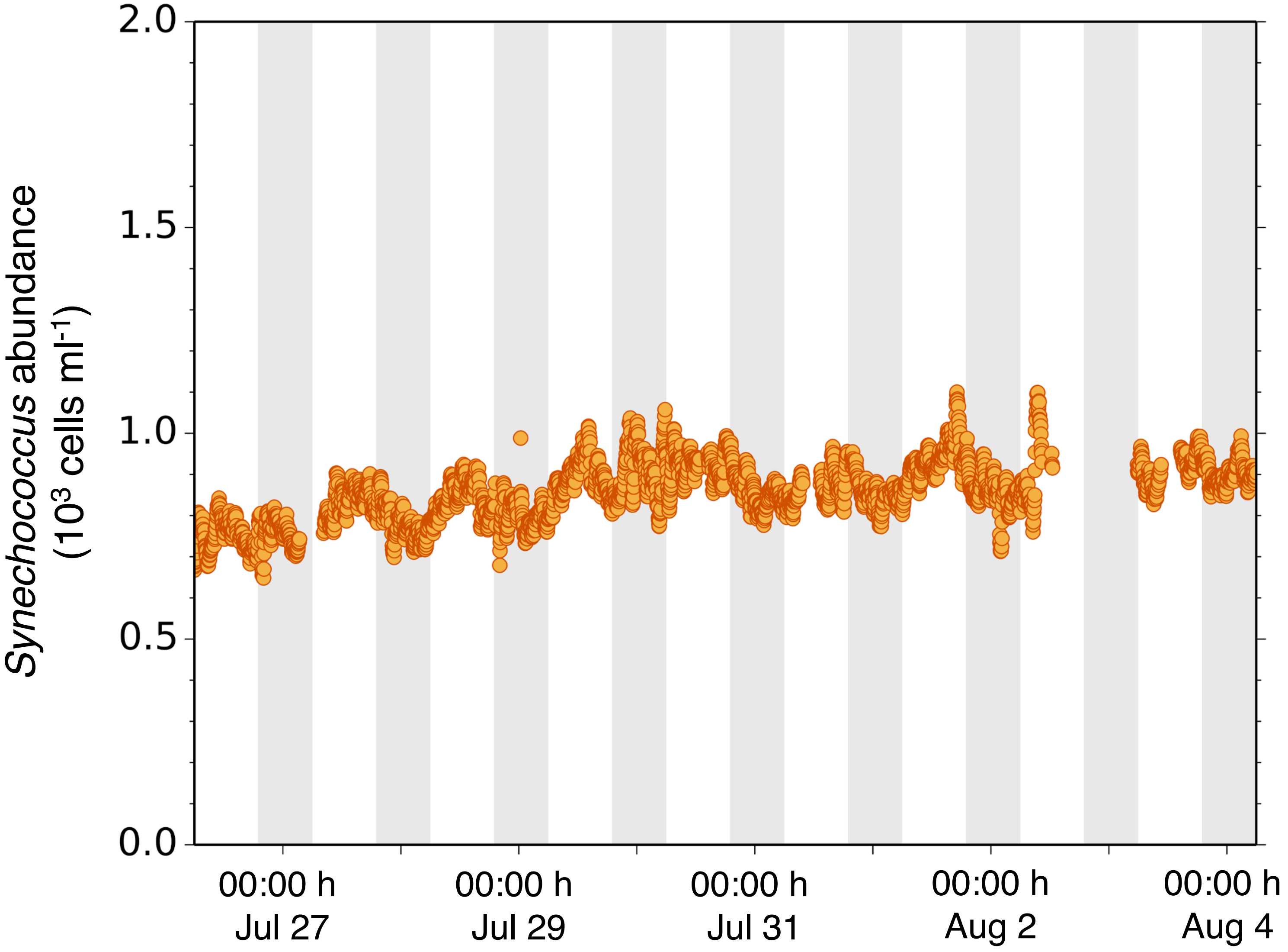

Supplementary Fig. 5. Abundances of *Synechococcus* at 5 m depth following a Lagrangian water mass in the North Pacific Subtropical Gyre in summer of 2015. Dark shaded regions indicate nighttime hours.

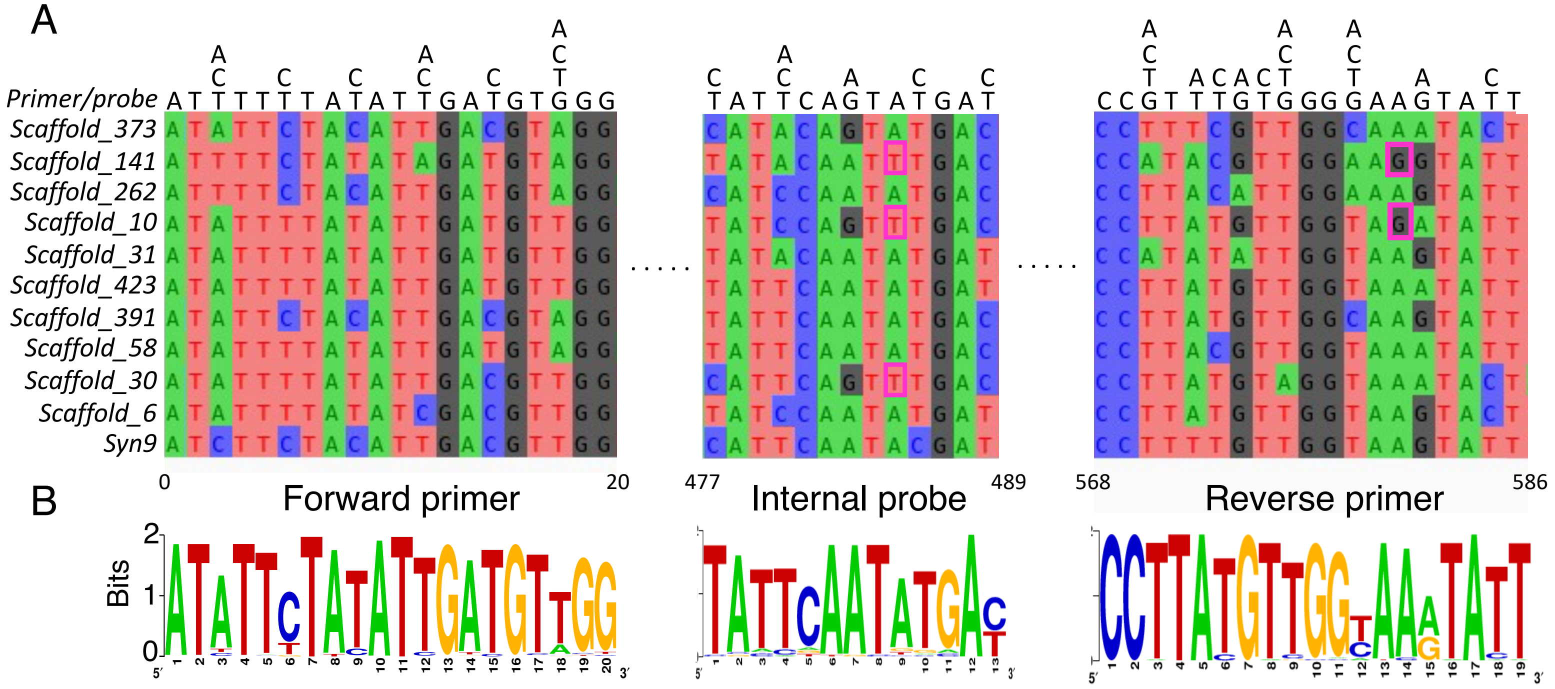

Supplementary Fig. 6. Comparison of environmental T4-like cyanophage *g20* sequences to degenerate primers and probes. (**a**) The alignment shows the regions of the *g20* gene that are targeted by the degenerate primers and probes used in the T4-like cyanophage polony method (34). The top row is the primer and probe sequences (sequences shown for the probe and reverse primer are the reverse complements). Degenerate bases in the primer/probe sequence are shown by stacked nucleotides. Cyanophage scaffolds assembled from viromes collected on the same 2015 cruise and a reference cultured T4-like cyanophage (Syn9) are shown below. Pink boxes highlight bases that are nucleotide mismatches to the primer and probe sequences. Note that single nucleotide mismatches were observed for 5 sequences and these were in the middle of the reverse primer and probe which would not interfere with amplification or detection. Empirical testing of the probe indicated that it could tolerate 3 mismatches (Goldin, personal communication). (**b**) Sequence logos show the relative frequency of bases at each position in the primers and probe based on the alignment of individual T4-like cyanophage reads from the 2015 viromes. The percent of reads aligned to the forward and reverse primers with two or less mismatches was 94% and 97%, respectively. Ninety three percent of reads aligned to the probe sequence with 3 or less mismatches.

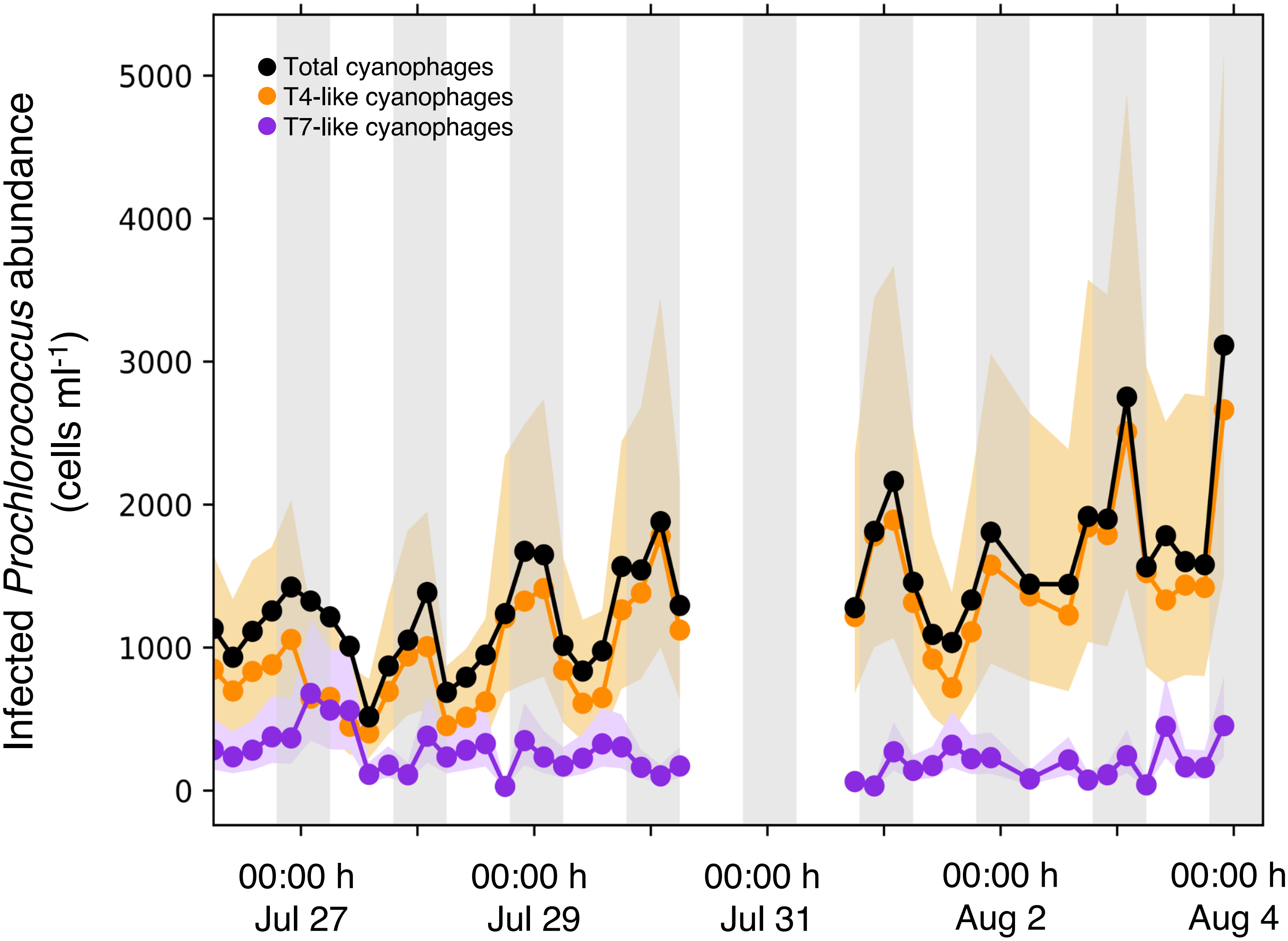

Supplementary Fig. 7. Diel periodicity of the number of infected *Prochlorococcus* cells in the North Pacific Subtropical Gyre in summer of 2015. Infection by T4-like (orange), T7-like (purple), and total (black) cyanophages is shown. Values (average and bounds) were derived from multiplying infection percentages by the abundance of *Prochlorococcus* at each time point. Gray bars indicate nighttime hours.

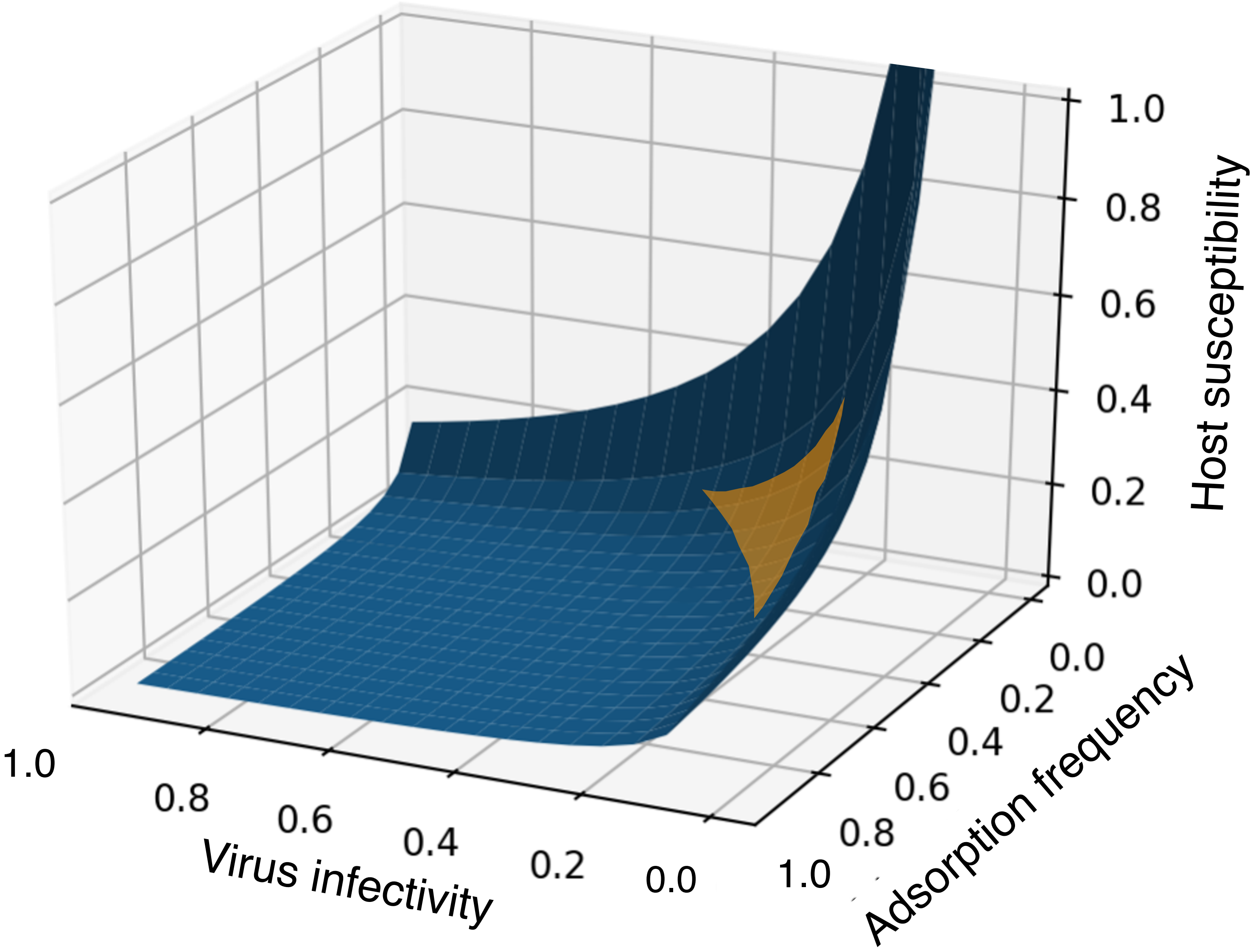

Supplemental Fig. 8. Solution space for the frequencies of mechanisms that are likely to mitigate infectious encounters. The plotted function shows all possible combinations of host susceptibility, virus infectivity, and adsorption efficiency that could explain the average 0.79% of encounters reported here to result in infection. Orange shading represents the region where all three parameters: adsorption frequency, virus infectivity and host susceptibility are balanced between 10-35% at the vertex of this space, assuming that no one parameter dominates as the mitigating factor. These values are within realistic ranges based on previously reported literature values for adsorption frequency and virus infectivity (see Supplementary results and discussion).

References for supporting information

73. Maruyama F, Tani K, Kenzaka T, Yamaguchi N, Nasu M. Quantitative determination of free-DNA uptake in river bacteria at the single-cell level by *in situ* rolling-circle amplification. Appl Environ Microbiol. 2006;72(9):6248-56.
74. Fuhrman JA, Comeau DE, Hagström A, Chan AM. Extraction from natural planktonic microorganisms of DNA suitable for molecular biological studies. Appl Environ Microbiol. 1988;54(6):1426-9.
75. Wegrzyn G, Wegrzyn A. Stress responses and replication of plasmids in bacterial cells. Microb Cell Fact. 2002;1(1):2-.
76. Kashtan N, Roggensack SE, Berta-Thompson JW, Grinberg M, Stepanauskas R, Chisholm SW. Fundamental differences in diversity and genomic population structure between Atlantic and Pacific *Prochlorococcus*. The ISME Journal. 2017;11(9):1997-2011.
77. Suttle CA, Chan AM. Marine cyanophages infecting oceanic and coastal strains of *Synechococcus* - Abundance, morphology, cross-infectivity and growth-characteristics. Mar Ecol Prog Ser. 1993;92(1-2):99-109.
78. Dekel-Bird NP, Sabehi G, Mosevitzky B, Lindell D. Host-dependent differences in abundance, composition and host range of cyanophages from the Red Sea. Environ Microbiol. 2015;17(4):1286-99.
79. Hanson CA, Marston MF, Martiny JBH. Biogeographic variation in host range phenotypes and taxonomic composition of marine cyanophage isolates. Front Microbiol. 2016;7:983-.
80. Flores CO, Meyer JR, Valverde S, Farr L, Weitz JS. Statistical structure of host–phage interactions. PNAS. 2011.
81. Weitz JS, Poisot T, Meyer JR, Flores CO, Valverde S, Sullivan MB, et al. Phage bacteria infection networks. Trends Microbiol. 2013;21(2):82-91.
82. Moore LR, Coe A, Zinser ER, Saito MA, Sullivan MB, Lindell D, et al. Culturing the marine cyanobacterium *Prochlorococcus*. Limnol Oceanogr Methods. 2007;5:353-62.
83. Morris JJ, Kirkegaard R, Szul MJ, Johnson ZI, Zinser ER. Facilitation of robust growth of *Prochlorococcus* colonies and dilute liquid cultures by “helper” heterotrophic bacteria. Appl Environ Microbiol. 2008;74(14):4530-4.
84. Zinser ER, Coe A, Johnson ZI, Martiny AC, Fuller NJ, Scanlan DJ, et al. *Prochlorococcus* ecotype abundances in the North Atlantic Ocean as revealed by an improved quantitative PCR method. Appl Environ Microbiol. 2006;72(1):723-32.
85. Menzel P, Ng KL, Krogh A. Fast and sensitive taxonomic classification for metagenomics with Kaiju. Nat Comm. 2016;7:11257.
86. Crummett LT, Puxty RJ, Weihe C, Marston MF, Martiny JBH. The genomic content and context of auxiliary metabolic genes in marine cyanomyoviruses. Virology. 2016;499:219-29.
87. Labrie SJ, Frois-Moniz K, Osburne MS, Kelly L, Roggensack SE, Sullivan MB, et al. Genomes of marine cyanopodoviruses reveal multiple origins of diversity. Environ Microbiol. 2013;15(5):1356-76.
88. Lukashin AV, Borodovsky M. GeneMark.hmm: New solutions for gene finding. Nucleic Acids Res. 1998;26(4):1107-15.
89. Edgar RC. MUSCLE: multiple sequence alignment with high accuracy and high throughput. Nucleic Acids Res. 2004;32(5):1792-7.

90. Larsson A. AliView: a fast and lightweight alignment viewer and editor for large datasets. Bioinformatics. 2014;30(22):3276-8.
91. Stamatakis A. RAxML version 8: a tool for phylogenetic analysis and post-analysis of large phylogenies. Bioinformatics. 2014;30(9):1312-3.
92. Altschul SF, Madden TL, Schäffer AA, Zhang J, Zhang Z, Miller W, et al. Gapped BLAST and PSI-BLAST: a new generation of protein database search programs. Nucleic Acids Res. 1997;25(17):3389-402.
93. Berger SA, Stamatakis A. PaPaRa 2.0: A vectorized algorithm for probabilistic phylogeny-aware alignment extension. Heidelberg, Institute for Theoretical Studies. 2012.
94. Crooks GE, Hon G, Chandonia JM, Brenner SE. WebLogo: a sequence logo generator. Genome Res. 2004;14(6):1188-90.
95. Einstein A. Über die von der molekularkinetischen Theorie der Wärme geforderte Bewegung von in ruhenden Flüssigkeiten suspendierten Teilchen. Annalen der Physik 1905;322:549–60.
96. Weigele PR, Pope WH, Pedulla ML, Houtz JM, Smith AL, Conway JF, et al. Genomic and structural analysis of Syn9, a cyanophage infecting marine *Prochlorococcus* and *Synechococcus*. Environ Microbiol. 2007;9(7):1675-95.
97. Kimmance SA, Brussaard CPD. Estimation of viral-induced phytoplankton mortality using the modified dilution method. In: S. W. Wilhelm MGW, and C. A. Suttle, editor. Manual of Aquatic Viral Ecology: ASLO; 2010. p. 65-73.
98. Steward GF, Wikner J, Smith DC, Cochlan WP. Estimation of virus production in the sea. I: Method development. Mar Microb Food Webs. 1992;6:57-78.
99. Noble RT, Fuhrman JA. Rapid virus production and removal as measured with fluorescently labeled viruses as tracers. Appl Environ Microbiol. 2000;66(9):3790-7.
100. Tadmor AD, Ottesen EA, Leadbetter JR, Phillips R. Probing individual environmental bacteria for viruses by using microfluidic digital PCR. Science. 2011;333(6038):58-62.
101. Allers E, Moraru C, Duhaime M, Beneze E, Solonenko N, Canosa JB, et al. Single-cell and population level viral infection dynamics revealed by phageFISH, a method to visualize intracellular and free viruses. Environ Microbiol. 2013;15:2306-18.
102. Dang VT, Sullivan MB. Emerging methods to study bacteriophage infection at the single-cell level. Front Microbiol. 2014;5:724.
103. Roux S, Hawley AK, Torres Beltran M, Scofield M, Schwientek P, Stepanauskas R, et al. Ecology and evolution of viruses infecting uncultivated SUP05 bacteria as revealed by single-cell- and meta-genomics. Elife. 2014;3:e03125.
104. Munson-McGee JH, Peng S, Dewerff S, Stepanauskas R, Whitaker RJ, Weitz JS, et al. A virus or more in (nearly) every cell: ubiquitous networks of virus-host interactions in extreme environments. ISME J. 2018;12(7):1706-14.
105. Thompson LR, Zeng Q, Chisholm SW. Gene expression patterns during light and dark infection of *Prochlorococcus* by cyanophage. PLoS One. 2016;11(10):e0165375.
106. Fridman S, Flores-Uribe J, Larom S, Alalouf O, Liran O, Yacoby I, et al. A myovirus encoding both photosystem I and II proteins enhances cyclic electron flow in infected *Prochlorococcus* cells. Nat Microbiol. 2017;2(10):1350-7.
107. Doron S, Fedida A, Hernandez-Prieto MA, Sabehi G, Karunker I, Stazic D, et al. Transcriptome dynamics of a broad host-range cyanophage and its hosts. ISME J. 2016;10(6):1437-55.

108. Bailey S, Clokie MR, Millard A, Mann NH. Cyanophage infection and photoinhibition in marine cyanobacteria. Res Microbiol. 2004;155(9):720-5.
109. Puxty RJ, Evans DJ, Millard AD, Scanlan DJ. Energy limitation of cyanophage development: implications for marine carbon cycling. ISME J. 2018;12(5):1273-86.
110. Wang K, Chen F. Prevalence of highly host-specific cyanophages in the estuarine environment. Environ Microbiol. 2008;10(2):300-12.
